# Tumor factors stimulate lysosomal degradation of tumor antigens and undermine their cross-presentation in lung cancer

**DOI:** 10.1038/s41467-022-34428-w

**Published:** 2022-11-04

**Authors:** Zhen Lu, Jinyun Chen, Pengfei Yu, Matthew J. Atherton, Jun Gui, Vivek S. Tomar, Justin D. Middleton, Neil T. Sullivan, Sunil Singhal, Subin S. George, Ashley G. Woolfork, Aalim M. Weljie, Tsonwin Hai, Evgeniy B. Eruslanov, Serge Y. Fuchs

**Affiliations:** 1 Department of Biomedical Sciences, School of Veterinary Medicine, University of Pennsylvania, Philadelphia, PA 19104 USA; 2 Department of Clinical Sciences & Advanced Medicine, School of Veterinary Medicine, University of Pennsylvania, Philadelphia, PA 19104 USA; 3 Department of Biological Chemistry and Pharmacology, The Ohio State University, Columbus, OH 43210 USA; 4 Division of Thoracic Surgery, Department of Surgery, Perelman School of Medicine, University of Pennsylvania, Philadelphia, PA 19104 USA; 5 Institute for Biomedical Informatics, Perelman School of Medicine, University of Pennsylvania, Philadelphia, PA 19104 USA; 6 Department of Systems Pharmacology and Translational Therapeutics, Perelman School of Medicine, University of Pennsylvania, Philadelphia, PA 19104 USA

**Keywords:** Tumour immunology, Cancer microenvironment, Non-small-cell lung cancer, Conventional dendritic cells, Antigen-presenting cells

## Abstract

Activities of dendritic cells (DCs) that present tumor antigens are often suppressed in tumors. Here we report that this suppression is induced by tumor microenvironment-derived factors, which activate the activating transcription factor-3 (ATF3) transcription factor and downregulate cholesterol 25-hydroxylase (CH25H). Loss of CH25H in antigen presenting cells isolated from human lung tumors is associated with tumor growth and lung cancer progression. Accordingly, mice lacking CH25H in DCs exhibit an accelerated tumor growth, decreased infiltration and impaired activation of intratumoral CD8^+^ T cells. These mice do not establish measurable long-term immunity against malignant cells that undergo chemotherapy-induced immunogenic cell death. Mechanistically, downregulation of CH25H stimulates membrane fusion between endo-phagosomes and lysosomes, accelerates lysosomal degradation and restricts cross-presentation of tumor antigens in the intratumoral DCs. Administration of STING agonist MSA-2 reduces the lysosomal activity in DCs, restores antigen cross presentation, and increases therapeutic efficacy of PD-1 blockade against tumour challenge in a CH25H-dependent manner. These studies highlight the importance of downregulation of CH25H in DCs for tumor immune evasion and resistance to therapy.

## Abstract (web summary)

Dendritic cells (DC) present tumour antigens to T cells but this process is defective in the tumour microenvironment. Here the authors find that downregulation of cholesterol 25-hydroxylase underlies defective cross presentation of tumour antigens.

## Introduction

Lung cancer remains one of the leading causes of cancer-related death^1^, thus new strategies for treatment of lung cancer are urgently needed. To date, immunotherapies directed toward boosting host antitumor T cell immunity are at the forefront of cancer therapeutics. However, despite recent successes with checkpoint blockade, these immunotherapies often fail to induce a durable antitumor response in a substantial percentage of patients with lung cancer^2^. Poor immunogenicity of tumor antigens and immune suppressive effects of the tumor microenvironment that impedes cross-presentation of these antigens by professional antigen presenting cells (APC, including conventional type 1 DCs) represent major barriers to effective adaptive anti-tumor immunity and efficient immune therapies. Importantly, emerging evidence from studies in mouse models and human patients suggests a possibility to interfere with immune suppressive effects of the tumor microenvironment and improve the anti-tumorigenic function of APC^3–5^.

Understanding the mechanisms underlying inhibition of APC function and tumor antigen presentation (reviewed in refs. 6, 7) is of critical importance for developing means for APC reactivation. These mechanisms include inhibition of DC recruitment, survival or maturation^8–14^. In addition, the tumor microenvironment-derived factors (TMDF) also impede the ability of DCs to present tumor antigens. For example, factors produced by tumors such as vascular endothelial growth factor (VEGF^14^) or prostaglandin E2 (PgE_2_^11,15,16^) were shown to inhibit antigen cross-presentation by DCs.

Tumor-infiltrating DCs take up elements from malignant cells before migrating to draining lymph nodes. There, DCs cross-present tumor antigens processed and loaded onto MHC-I molecules to prime cytotoxic CD8^+^ T cells (reviewed in refs. 7, 10, 17, 18). While cross-presentation obviously requires some lysosomal activity in DCs, this activity has to be carefully regulated to prevent a scenario wherein internalized antigens undergo rapid proteolysis, which can inhibit cross-presentation^19,20^. Indeed, tightly controlled and restricted proteolysis of antigens was implicated in efficient cross-presentation by migratory DCs^20,21^. Furthermore, therapeutic use of lysosomal inhibitors can improve antigen presentation^22^. Here we show that conditions of tumor microenvironment and TMDF act to increase lysosomal degradation of antigens and attenuate their cross-presentation in DCs in a manner dependent on the upregulation of activating transcription factor-3 (ATF3) and downregulation of cholesterol 25-hydroxylase (CH25H).

*ATF3* is a stress-inducible gene, which encodes an important transcription factor that is involved in development and progression of a number of human cancers as either pro-tumorigenic or anti-tumorigenic, depending on the context^23^. Overexpression of ATF3 noted in NSCLC^24^ was associated with accelerated tumor progression and poor prognosis^25^. Deletion of ATF3 in malignant cells decrease their ability to produce PD-L1 checkpoint ligand^26^. In breast cancers, ATF3 expression in monocytic myeloid cells was shown to contribute to tumor growth, progression and metastases^27,28^. Here we demonstrate that expression of ATF3 in DCs has an important function in stimulating tumor growth.

We found that *ATF3* inhibits the expression of *CH25H* in DCs. The activities of *CH25H*, which is an interferon-stimulated gene^29^, in tumors in general and lung cancers specifically remains largely unknown. This enzyme catalyzes production of 25-hydroxycholesterol (25HC), which suppresses cholesterol synthesis and affects several important immune pathways^30^. 25HC also acts to inhibit fusion of lipid membranes^31–33^; this property of 25HC is likely to support its activity in suppressing the uptake of the tumor-derived extracellular vesicles, which promote intratumoral angiogenesis and metastasis^33,34^.

In this work, we show that factors present in the tumor microenvironment induce ATF3 and decrease the levels of CH25H in the intratumoral DCs. In turn, downregulation of CH25H in the intratumoral DCs leads to augmented lysosomal proteolysis of antigens and results in an impaired antigen cross presentation and accelerated tumor growth.

## Results

### ATF3 expression in DCs promotes tumor growth

**Fig. 1:**
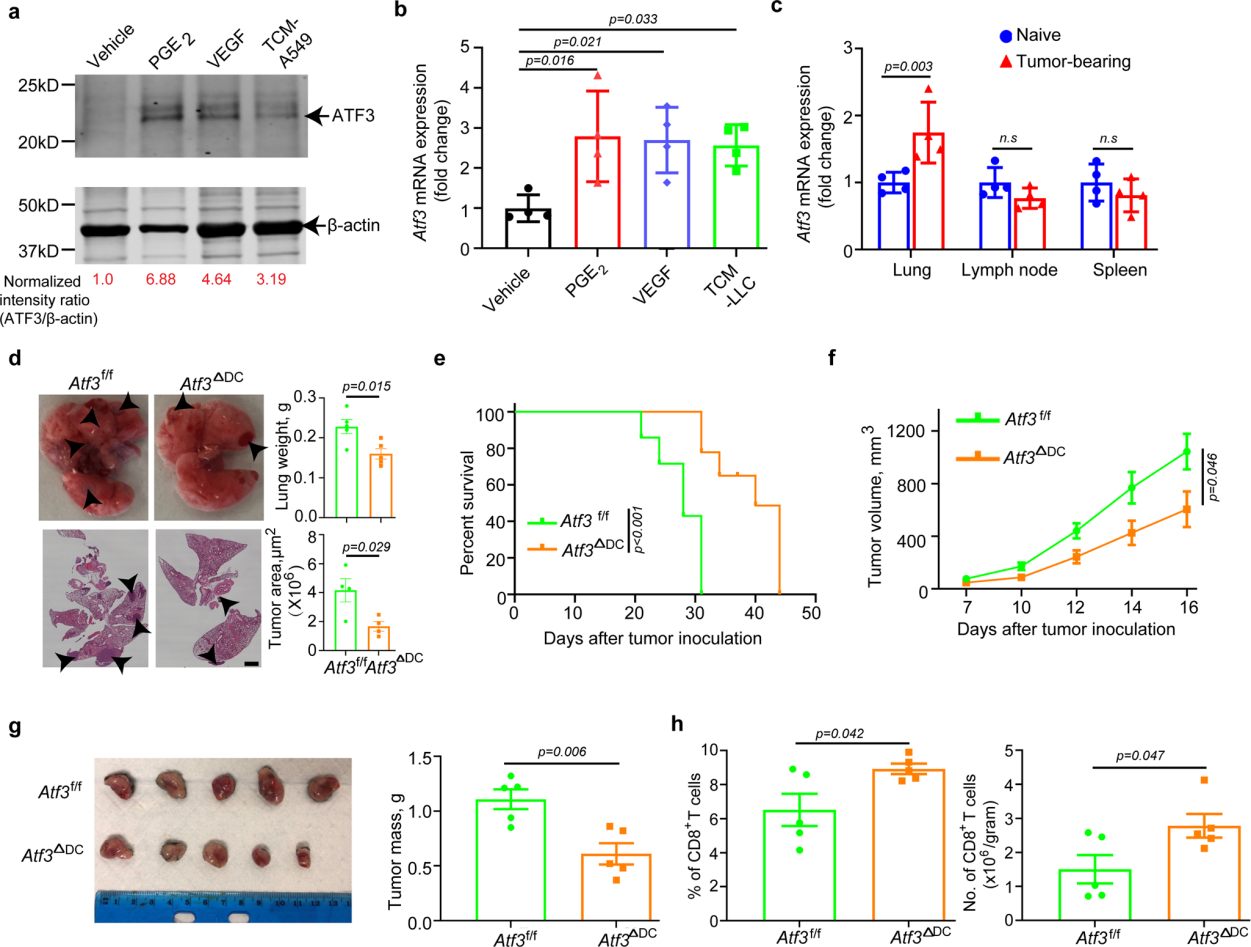
Tumor microenvironment factors-induced ATF3 in DCs promotes tumor growth. **a** Immunoblot analysis of ATF3 levels in human monocytes treated with vehicle, Prostaglandin E_2_ (PGE_2,_ 10 ng/mL), vascular endothelial growth factor (VEGF, 20 ng/mL), or tumor cell-conditioned media (TCM—from A549 cells, 75%, v/v) for 1.5 h. Levels of β-actin (as a loading control) are also shown. **b** qPCR analysis of *Atf3* mRNA in mouse splenic CD11c^+^ myeloid cells treated with vehicle, PGE_2_ (10 ng/mL), VEGF (20 ng/mL) or TCM from Lewis lung carcinoma cells (LLC, 75%, v/v) for 2 h. *n* = 4 biologically independent samples. **c** qPCR analysis of *Atf3* mRNA levels in DCs isolated from lungs, lung-draining lymph nodes (LNs) and spleens from either naïve or lung LLC-bearing mice (inoculated *i.v*., 1 × 10^6^ cells/mouse, 2 weeks before isolation and analysis). *n* = 4 biologically independent samples. **d** Tumor weight, representative lung images and the corresponding H&E-stained lung sections from *Atf3*^f/f^ and *Atf3*^ΔDC^ groups (*n* = 4 mice per group) 14 days after intravenous injection of 6 × 10^5^ LLC tumor cells. Scale bar:2 mm. Similar results were obtained from three independent experiments. **e** Kaplan–Meier analysis of survival of LLC tumor-bearing mice (after intravenous injection of 4.5 × 10^5^ LLC cells) by log-rank test. *n* = 7 mice in both *Atf3*^f/f^ and *Atf3*^ΔDC^ groups. **f** Growth of LLC tumors after subcutaneous injection of 6.25 × 10^5^ LLC tumor cells into *Atf3*^f/f^ or *Atf3*^ΔDC^ mice. *n* = 5 mice in each group. **g** Representative images and quantification of LLC tumor mass at day 18 from the experiment described in panel **f**. **h** Flow-cytometric determination of the percentage and quantitative estimates of intratumoral CD8^+^ T cells from the experiment described in panel 1 f. *n* = 5 tumors in each group. Data are presented as mean ± SEM. Statistical analysis was performed using 2-tailed Students’ *t*-test (B, C, D, G and H) or 2-way ANOVA with multiple-comparison test (F) or Kaplan-Meier survival analysis (E) test. n.s., not significant. Source data are provided as a Source Data file.

We have previously demonstrated that *ATF3* is upregulated in the intratumoral monocytic cells in human cancers, and high levels of ATF3 in this compartment are associated with poor prognosis^27^. Intriguingly, we found that levels of ATF3 in human monocytic cells was modestly increased in vitro by the TMDF previously shown to interfere with DC function such as VEGF^14^ and PGE_2_^11,15,16^. Similar results were obtained using media conditioned by cultured human lung cancer cells (Fig. 1a, Supplementary Fig. 1a). Given that gene expression signatures regulated by ATF3 included those involved in cell-mediated immune response and antigen presentation^27^, we next focused on the status and functions of ATF3 in conventional DCs. These cells express the CD11c integrin, which, although not unique, is widely used as the means for mouse DC purification and lineage-specific genetic manipulations^35,36^. Accordingly, as accepted in the field, here we used the term “DCs” for mouse CD11c^+^ cells.

In mouse DCs, ATF3 expression was induced in vitro by VEGF, PGE_2_ and media conditioned by Lewis lung carcinoma (LLC) cells (Fig. 1b). Furthermore, greater levels of ATF3 expression were found in DCs isolated from the lungs of mice that bear pulmonary LLC tumors compared to DCs isolated from naïve lungs. This difference was not seen in DCs isolated from either lymph nodes or spleens (Fig. 1c) indicating that factors present in the tumor microenvironment induce ATF3 in DCs.

To examine the biological importance of ATF3 expression in DCs in the context of pulmonary tumor growth, we have inoculated LLC cells intravenously into *Atf3*^f/f^ or Cd11c-Cre; *Atf3*^f/f^ (hereafter termed *Atf3*^ΔDC^) mice (Supplementary Fig. 1b, c). No notable differences in frequency of splenic CD8^+^ T cells, DCs and their expression of CD80 marker were observed in these animals (Supplementary Fig. 1d). We found that mice lacking ATF3 in DCs exhibited a decreased pulmonary tumor burden as manifested by macroscopic observations, and quantification of lung weight and tumor lesions (Fig. 1d). Furthermore, ablation of ATF3 in DCs exhibited a prolonged animal survival (Fig. 1e). This inhibition of LLC tumor growth in mice, whose DCs were deficient in ATF3, was further recapitulated in the subcutaneous tumor settings (Fig. 1f, g). Subcutaneous tumors from *Atf3*^ΔDC^ mice exhibited a trend for somewhat greater numbers of DCs (Supplementary Fig. 1e) and significant increase in the numbers and frequency of CD8^+^ T cells compared with tumors from control animals (Fig. 1h). These results collectively suggest that ATF3 expression in DCs contributes to stimulating tumor growth and attenuating the anti-tumor immune responses.

### CH25H is downregulated in intratumoral DCs; low levels of CH25H are associated with tumor growth and progression

**Fig. 2:**
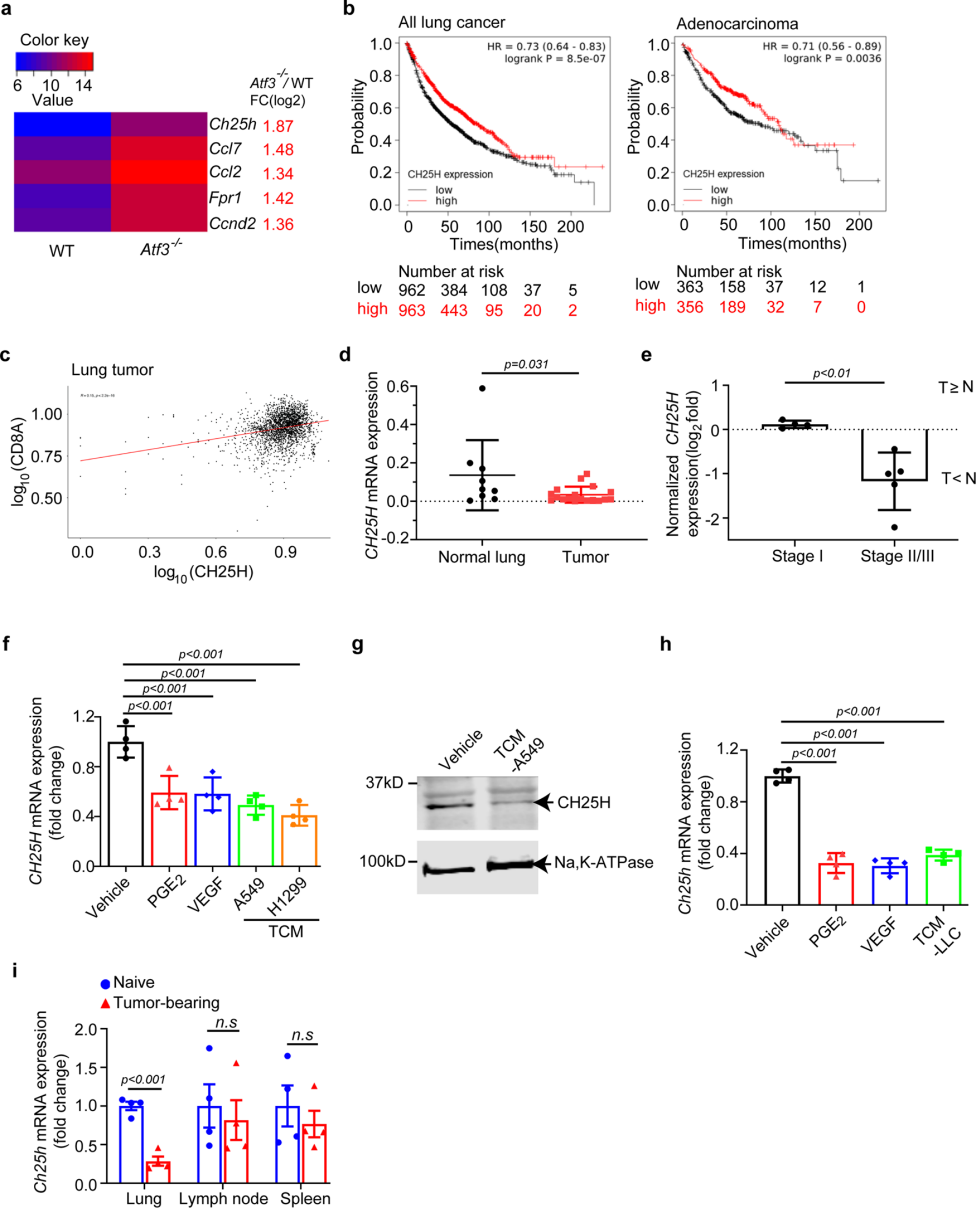
Downregulation of CH25H in human and mouse lung tumors. **a** A heat map for the top five differentially expressed genes in an Agilent Whole Mouse Genome Microarray using RNA isolated from WT or *Atf3* ^−/−^ bone marrow-derived macrophages. Values are the average log_2_ (intensity) of the signal from probes against the indicated genes (*n* = 4 mice per genotype). *Ch25h*: cholesterol 25-hydroxylase; *Ccl7*: C-C motif chemokine ligand 7; *Ccl2*: C-C motif chemokine ligand 7; *Fpr1*: formyl peptide receptor 1; *Ccnd2*: cyclin D2. **b** Overall survival curves for *CH25H* expression (Affy ID: 206932_at) in tumors from patients with non-small cell lung cancers (NSCLC) or lung adenocarcinomas were generated in KM plotter using default parameters and no restrictions. Patients split by median survival. **c** Correlation analysis between expression of *CH25H* and of marker for CD8^+^ T cell presence *CD8A* in the human lung cancer tumor samples from the GENT2 public database. The plot was generated using the graphical and statistical software R. **d** qPCR analysis of *CH25H* expression in CD14^+^ cells from primary lung tumors and distant (“Normal”) lung tissues from NSCLC patients. *n* = 18 tumors and *n* = 9 distant lungs. **e** Clinical stages and *CH25H* expression of NSCLC patients. The Fisher’s exact test was used to analyze the correlation between *CH25H* expression and clinical stages. T ≥ N, comparable *CH25H* levels within CD14^+^ monocytes from tumor and distant normal lungs; T < *N*, lower level of *CH25H* in intratumoral CD14^+^ monocytes compared to cells isolated from distant normal lungs. *n* = 4 for stage I and *n* = 5 for stage II/III lung cancer. **f** qPCR analysis of *CH25H* mRNA levels in human monocytes treated with vehicle, PGE_2_ (10 ng/mL), VEGF (20 ng/mL), or TCM media from A549 or H1299 cells (75%, v/v). *n* = 4 biologically independent samples for each group. **g** Immunoblotting analysis of CH25H protein in human monocytes treated with control medium and medium conditioned from A549 cells (75%, v/v) for 3 h. Levels of Na,K-ATPase that serves as a loading control are also shown. **h** qPCR analysis of *Ch25h* mRNA in mouse CD11c^+^ splenic myeloid cells treated with vehicle, PGE_2_ (10 ng/mL), VEGF (20 ng/mL) or medium conditioned from LLC cells (75%, v/v). *n* = 4 biologically independent samples for each group. **i** qPCR analysis of *Ch25h* mRNA in DCs isolated and purified from tumor lung, lung-draining LNs and spleen from naïve mice and LLC inoculated mice (i.v., 1 × 10^6^ cells/mouse, 2 weeks after inoculation). *n* = 4 mice per group. Data are presented as mean ± SEM. Statistical analysis was performed using 2-tailed Students’ *t*-test (D, E, F, H and I) or KM plotter (B) test. n.s., not significant. Source data are provided as a Source Data file.

As a first step to investigate the downstream target genes of ATF3 that may contribute to the phenotype, we carried out microarray analysis of bone marrow derived macrophages from WT or *Atf3* ^−/−^ mice. We used these macrophages because, similar to DCs, they are also APCs. Furthermore, these cells can be obtained in relatively high quantity. Examination of gene expression profiles in ATF3-deficient APC showed *CH25H* amongst the top 5 genes upregulated upon ATF3 ablation (Fig. 2a). This result is consistent with previously demonstrated functions of ATF3 in suppressing transcription of *Ch25h* in mouse macrophages^37^.

To test whether this differential expression occurs in DCs, we isolated CD11c^+^ splenic myeloid cells from *Atf3*^f/f^ and *Atf3*^ΔDC^ mice and found that CH25H is upregulated in *Atf3*-null DCs but not in CD11c- cells (Supplementary Fig. 2a). Importantly, analysis of genes, whose expression was downregulated in the intratumoral conventional type 1 DCs compared to these cells isolated from apparently benign tissues from the same patients with breast cancers also implicated *CH25H* as one of these genes^38^. In our own studies, we next focused on *CH25H* expression and its importance in DCs in lung cancer.

Overall, analysis of published expression and survival data obtained from patients with non-small cell lung cancer and lung adenocarcinoma^39^ showed that low *CH25H* expression was associated with poor prognosis in these patients (Fig. 2b, Supplementary Fig. 2b). Furthermore, in tumors from patients with lung cancers^40^, *CH25H* expression positively correlated with levels of *CD8A* (Fig. 2c) indicating a putative link between CH25H and the presence of cytotoxic CD8^+^ tumor infiltrating lymphocytes within the tumor microenvironment.

It was not feasible to isolate cDC1 from human lung tumors due to very low frequency of these cells (<1%). Thus, we isolated cells expressing CD14, a marker for myeloid APC (including DCs, monocytes and macrophages) for the analysis of CH25H gene expression. We isolated the cells from non-small cell lung tumors or from distant lung parenchyma (within the same lobe) of patients (*N* = 18) with early lung cancers^5,41^. Although the values were highly variable, we detected a significant decrease in *CH25H* expression in CD14^+^ cells isolated from tumors compared to those from distant lung tissues (Fig. 2d). Importantly, double blind pairwise analysis of tissues from these patients outlined two distinct groups. One of these groups was characterized by a decrease in *CH25H* expression in the intratumoral CD14^+^ cells compared to their counterparts isolated from distant lung tissues. The other group showed no change or even a modest increase in *CH25H* mRNA levels (Supplementary Fig. 2c). Intriguingly, analysis of clinical data indicated that CD14^+^ cells exhibiting a decrease in *CH25H* expression in tumors originated from patients with more advanced disease (Fig. 2e, Supplementary Fig. 2d). These results link downregulation of CH25H in the intratumoral CD14^+^ cells with growth and progression of human lung cancers.

In line with these data, normal human monocytes isolated from peripheral blood of healthy donors notably downregulated CH25H after in vitro treatment with TMDF (Fig. 2f, g). These data were recapitulated in mouse DCs wherein TMDF also downregulated CH25H expression in vitro (Fig. 2h). Furthermore, lower expression of CH25H was detected in DCs isolated from the lungs but not spleens or lymph nodes of LLC pulmonary tumors-bearing mice compared to DCs from the lungs of naïve mice (Fig. 2i). In all, these results suggest that factors present in the tumor microenvironment downregulate CH25H expression in intratumoral DCs.

### CH25H enables efficient antigen cross-presentation expression in DCs

**Fig. 3:**
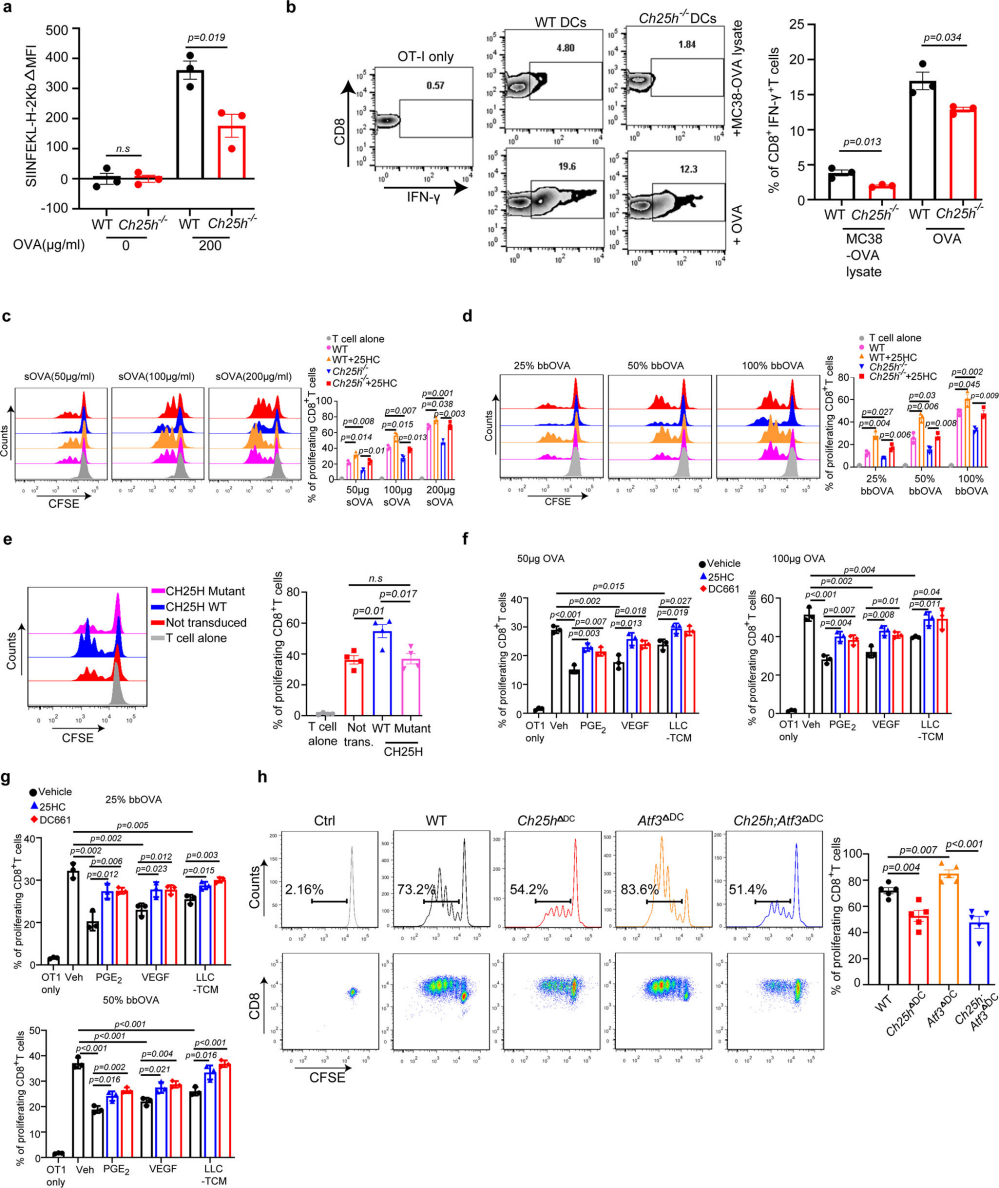
CH25H expression in DCs enables efficient antigen cross-presentation otherwise inhibited by factors of tumor microenvironment. **a** Merged MFI expression of SIINFEKL-bound H-2kb in DCs from indicated mice after treatment of OVA protein (200 μg/mL, 18 h). *n* = 3 biologically independent samples. **b** Percentage of IFNγ^+^ in OT-I CD8^+^ T cells after 72 h co-culture of naïve OT-I CD8^+^ T cells with splenic CD11c^+^ myeloid cells from indicated mouse pulsed with OVA (10:1) or MC38-OVA lysate (10:4). *n* = 3 biologically independent samples. **c** Antigen cross presentation analysis for WT or *Ch25h*^−/−^ DCs pre-treated or not with 25HC (50 nM, 4 h before adding soluble sOVA at indicated concentrations). OVA-pulsed DCs were then co-cultured (10:1 for 72 h) with OT-I CD8^+^ T cells labeled with carboxy fluorescein succinimidyl ester (CFSE). Proliferation of these T cells was assessed by CFSE dilution. *n* = 3 biologically independent samples. **d** Antigen cross presentation analysis for WT or *Ch25h*^−/−^ DCs pre-treated or not with 25HC (50 nM, 4 h before adding beads loaded with OVA at indicated percentage) was carried out as in panel C. *n* = 3 biologically independent samples. **e** Antigen cross presentation analysis for *Ch25h*^−/−^ DCs transduced with retroviruses for expression of CH25H^WT^ or catalytically inactive CH25H^H242,243Q^ mutant. *n* = 4 biologically independent samples. **f** Antigen cross presentation analysis for WT DCs pre-treated with or without PGE_2_ (10 ng/mL), VEGF (20 ng/mL) or TCM from LLC cells (75%, v/v) for 24 h with or without the treatment of 25HC (50 nM) or DC661 (5 μM) for 4 h as indicated. Then DCs were pulsed with soluble OVA protein (50 or 100 μg/mL, 6 h) and co-cultured with CFSE-labeled OT-I T cells (10:1 for 72 h). *n* = 3 biologically independent samples. **g** Antigen cross presentation analysis for WT DCs pre-treated with or without PGE_2_ (10 ng/mL), VEGF (20 ng/mL) or TCM from LLC cells (75%, v/v) for 24 h with or without the treatment of 25HC (50 nM) or DC661 (5 μM) for 4 h as indicated. Then DCs were then treated with beads-bound OVA protein (25% or 50%, 1 h) and co-cultured with CFSE-labeled OT-I T cells (10:1 for 72 h). *n* = 3 biologically independent samples. **h** Antigen cross presentation analysis for DCs of indicated genotypes. T cell proliferation was assessed by flow cytometry in CFSE-labeled OT-I T cells co-cultured (10:1 for 72 h) with DCs from indicated mice, pretreated with conditioned media from LLC cells (70%, v/v) for 24 h and pulsed with soluble OVA protein (200 μg/mL, 6 h). *n* = 5 biologically independent samples. **h** Data are presented as mean ± SEM. Statistical analysis was performed using 2-tailed Students’ *t*-test (A, B, C, D, E and H) or 1-way ANOVA with Tukey’s multiple-comparison test (F and G) test. n.s., not significant. Source data are provided as a Source Data file.

Given that TMDF downregulate CH25H, we next assessed the importance of this downregulation in DC functions. To this end, we compared the characteristics of DCs from wild type (*Ch25h*^+/+^, WT) and *Ch25h*^−/−^ knockout mice. We found that genetic ablation of CH25H did not affect expression of co-stimulatory CD80 and CD86 markers regardless of treatment with lipopolysaccharide (Supplementary Fig. 3a). WT and CH25H-deficient splenic DCs also displayed similar cell surface levels of MHC-I (Supplementary Fig. 3b) and comparable ability for uptake (Supplementary Fig. 3c) and direct presentation (Supplementary Fig. 3d) of minimal OVA-derived peptide antigen. However, when compared to WT DCs, CH25H-deficient cells were less competent in the OVA protein antigen cross-presentation as manifested by the levels of antigen-MHC-I complexes (Fig. 3a, Supplementary Fig. 3e) as well as by ability of these DCs to increase production of IFN-γ by CD8^+^ T cells (Fig. 3b) and to stimulate T cell proliferation when delivered to DCs as either soluble (Fig. 3c) or particle-bound (Fig. 3d) protein. Importantly, these phenotypes in *Ch25h*-deficient DCs were reverted by treating these cells with the enzymatic product of CH25H, 25HC (Fig. 3c, d). Furthermore, re-expression of wild type CH25H but not of its catalytically inactive CH25H^H242, 243Q^ mutant increased antigen cross presentation (Fig. 3e, Supplementary Fig. 3, f). Together, these data support the notion that the loss of CH25H and decrease in 25HC in DCs undermine antigen cross-presentation.

To further test the functional importance of downregulation of CH25H, we complemented the above data derived from genetically modified *Ch25h*^*−/−*^ DCs by using WT cells. We pre-treated the WT DCs with PGE2, VEGF or LLC tumor cell-conditioned media to downregulate CH25H (as in Fig. 2). Interestingly, the treatments significantly decreased the ability of these cells to present soluble (Fig. 3f, Supplementary Fig. 3g) or particle-bound (Fig. 3g, Supplementary Fig. 3g) OVA protein antigens, providing correlative evidence that downregulation of CH25H by TMDF suppresses antigen cross-presentation. Importantly, this suppression either in TMDF-treated WT DCs or in CH25H-null DCs was largely rescued by pre-treatment of these cells with 25HC (Fig. 3f, g, Supplementary Fig. 3g) further indicating the importance of CH25H in regulating antigen cross-presentation.

Phenotypes found in DCs isolated from conventional knockout *Ch25h*^−/−^ mice may be indirectly influenced by mechanisms conferred by CH25H in other cell types. Thus, we generated conditional knockout *Cd11c-Cre*;*Ch25h*^f/f^ (hereafter termed *Ch25h*^ΔDC^) mice, which lacked *Ch25h* expression specifically in Cd11c^+^ cells (Supplementary Fig. 3h). DCs isolated from these mice were notably deficient in cross-presentation of OVA antigens (Fig. 3h), indicating a cell-autonomous activity of CH25H for this DC function. Importantly, robust antigen cross-presentation by ATF3-deficient DCs was significantly suppressed by additional ablation of *Ch25h* (Fig. 3h). Collectively, these results indicate that ATF3-dependent decrease in the levels of CH25H in DCs undermines the ability of these cells for efficient antigen cross-presentation.

### CH25H regulates antigen lysosomal degradation

We next focused on delineating the mechanisms underlying suboptimal antigen cross-presentation upon the loss of CH25H. This defect in CH25H-deficient DCs could not be readily explained by differences in the status of co-stimulatory markers, MHC-I expression or antigen uptake. These data led us to contemplate the potential importance of DC lysosomal degradation of antigens because of several independent considerations. To begin with, lysosomes play an important and nuanced function in antigen presentation due to its proteolytic function. In addition, accelerated proteolysis of antigens in the lysosomal compartment is known to restrict cross-presentation (reviewed in^42^). Furthermore, 25HC was shown to alter the fluidity of lipid membranes and inhibit their ability for fusion^31,32^, which is essential for maturation and function of lysosomes^43,44^. Importantly, treatment of DCs with the lysosomal inhibitor DC661 partially restored antigen cross presentation otherwise inhibited by TMDF (Fig. 3f, g, Supplementary Fig. 3g). Moreover, treatment with DC661 increased the antigen presentation abilities of *Ch25h*-null DCs to the level of WT DCs (Supplementary Fig. 4a).

**Fig. 4:**
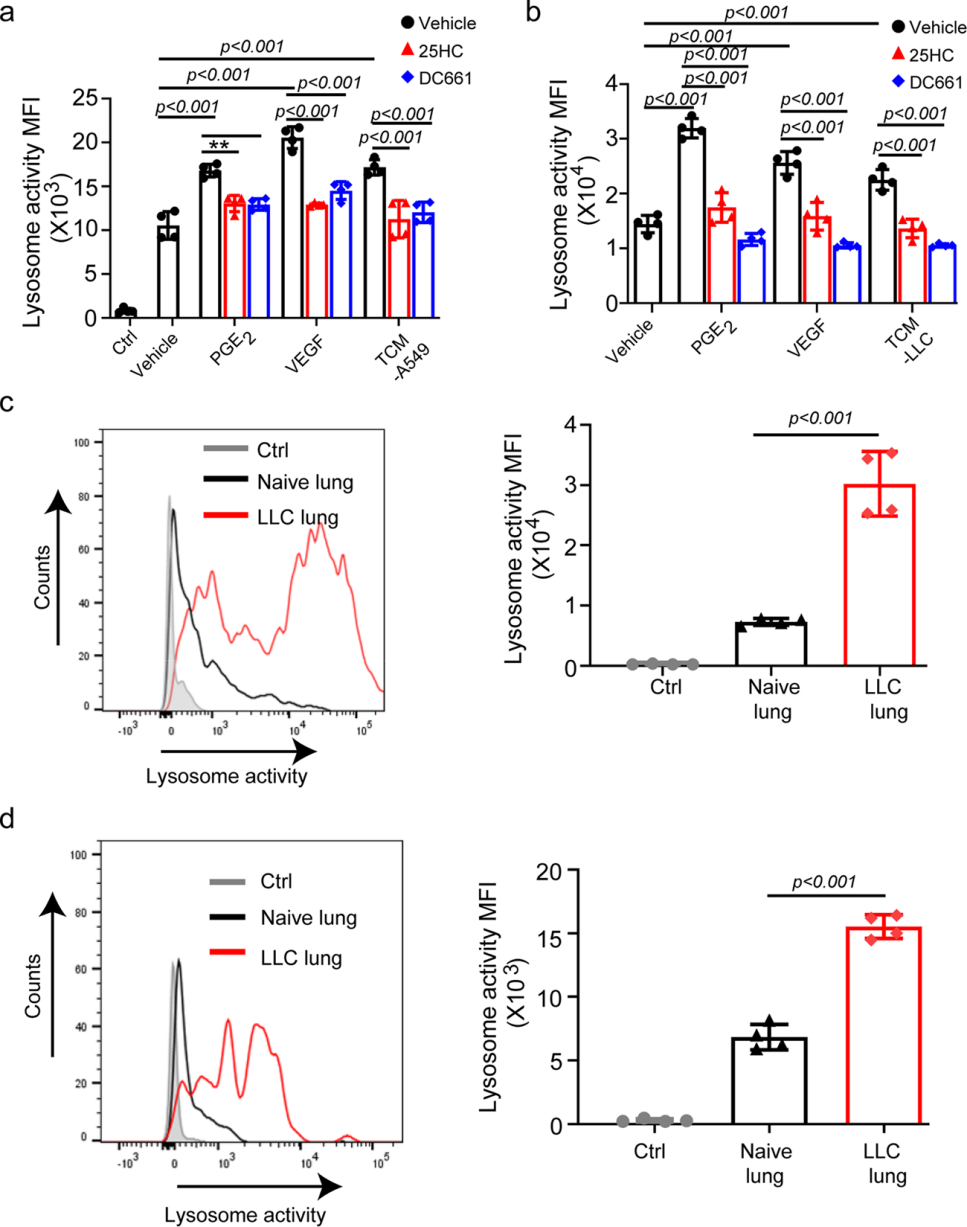
CH25H expression in DCs acts to limit the lysosomal degradation otherwise induced by factors of tumor microenvironment. **a** Quantitative analysis of DQ-OVA fluorescence in human CD14^+^ monocytes pretreated with PGE_2_ (10 ng/mL), VEGF (20 ng/mL) or TCM from A549 cells (75%, v/v) for 24 h with or without the treatment of 25HC (4 µM) or DC661 (100 nM). *n* = 4 biologically independent samples. **b** Quantitative analysis of DQ-OVA fluorescence in bone marrow derived DCs pretreated with PGE_2_ (10 ng/mL), VEGF (20 ng/mL) or TCM from LLC cells (75%, v/v) for 24 h with or without the treatment of 25HC (4 µM) or DC661 (100 nM). *n* = 4 biologically independent samples. **c** Representative histogram and quantitative analysis of DQ-OVA fluorescence in total DCs (CD45^+^CD11c^+^MHC II^+^) from naïve lungs or LLC tumor bearing lungs (*i.v*., 1 × 10^6^ cells/mouse, isolated 2 weeks after inoculation). Ctrl—DCs from LLC lung incubated without DQ-OVA substrate. *n* = 4 mice per group. **d** Representative histogram and quantitative analysis of DQ-OVA fluorescence in migratory DCs (CD45^+^ CD103^+^CD11b^-^) from naïve lungs or LLC tumor bearing lungs (*i.v*., 1 × 10^6^ cells/mouse, isolated 2 weeks after inoculation). Ctrl—DCs from LLC lung incubated without DQ-OVA substrate. *n* = 4 mice per group. Data presented as mean ± SEM. Statistical analysis was performed using 1-way ANOVA with Tukey’s multiple-comparison test (A and B) test or 2-tailed Students’ *t*-test (C and D). n.s., not significant. Source data are provided as a Source Data file.

Driven by these considerations, we assessed the effects of TMDF on the lysosomal proteolysis of antigens. Treatment with TMDF such as PGE_2_, VEGF or lung cancer cell-conditioned media notably stimulated lysosomal degradation of the conjugated DQ-OVA substrate in human monocytes in vitro (Fig. 4a, Supplementary Fig. 4b). A similar outcome was observed in mouse DCs (Fig. 4b, Supplementary Fig. 4c). In both settings, treatment with 25HC or a bona fide lysosomal inhibitor DC661 attenuated this increase in lysosomal activity to largely a comparable level (Fig. 4a, b, Supplementary Fig. 4b, c). These results suggest that downregulation of CH25H mediates the TMDF-stimulated increase in lysosomal degradation.

Importantly, greater lysosomal proteolytic activity was detected in DCs isolated from the lungs of mice that bear pulmonary LLC tumors compared to DCs isolated from naïve lungs (Fig. 4c). This difference was not seen when DCs were isolated from lymph nodes or spleens (Supplementary Fig. 4d). Similar results were obtained specifically for CD103^+^ migratory DCs (Fig. 4d, Supplementary Fig. 4e) indicating that factors present in the tumor microenvironment stimulate the lysosomal activity in intratumoral DCs in vivo.

**Fig. 5:**
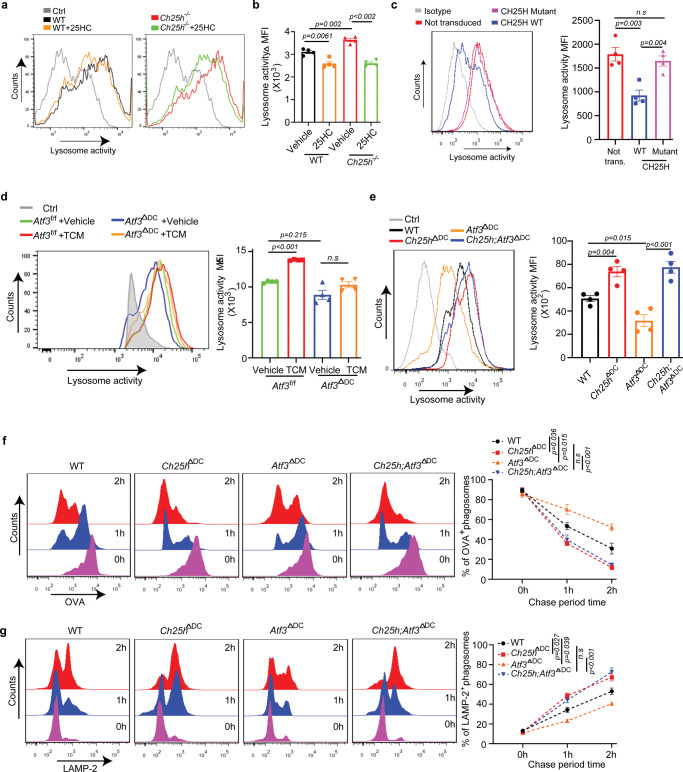
CH25H regulates lysosomal proteolysis. **a** Representative histogram of DQ-OVA fluorescence in DCs of indicated genotype pretreated with 25HC (4 μM, 4 h) as indicated. *n* = 4 biologically independent samples. **b** Quantification of data from panel **A. c** Representative histogram of DQ-OVA fluorescence in CH25H-null DCs transduced with retroviruses for expression of CH25H^WT^ or catalytically inactive CH25H^H242,243Q^ mutant. *n* = 4 biologically independent samples. **d** Representative histogram and quantitative analysis of DQ-OVA fluorescence in DCs isolated from Atf3^f/f^ or Atf3^ΔDC^ mice and pretreated with control media or TCM from LLC cells (67%, v/v) for 24 h. *n* = 3 biologically independent substrate-free group (Ctrl), *n* = 4 biologically independent experimental samples in experimental group. **e** Representative histogram and quantitative analysis of DQ-OVA fluorescence in indicated DCs pretreated with TCM from LLC cells (75%, v/v) for 24 h. *n* = 4 biologically independent samples. **f** Histograms show phagosomal degradation of OVA (left) and its quantification(right) after indicated chase periods (*n* = 3 biologically independent samples). **g** Histograms show phagosomal acquisition of LAMP2 (left) and its quantification (right) after indicated chase periods (*n* = 3 biologically independent samples). Data presented as mean ± SEM. Statistical analysis was performed using 1-way ANOVA with Tukey’s multiple-comparison test (B and D) test or 2-way ANOVA with multiple comparison (F and G) or 2-tailed Students’ *t*-test (C and E). n.s., not significant. Source data are provided as a Source Data file.

To complement these experiments, we used genetically modified DCs. Analysis of the lysosomal proteolytic activities in DCs using two different lysosomal OVA-based substrate reporters showed that CH25H-null DCs displayed a greater OVA degradation compared to WT counterparts (Fig. 5a, b, Supplementary Fig. 5a, b). Treatment with 25HC abrogated these differences (Fig. 5a, b). Furthermore, re-expression of CH25H^WT^ but not of inactive CH25H^H242,243Q^ mutant (Supplementary Fig. 3f) decreased lysosomal degradation (Fig. 5c). Consistent with the function of ATF3 in suppressing CH25H expression (Supplementary Fig. 2a), ATF3-null DCs exhibited a reduced OVA proteolysis, which was not increased after treatment with the lung cancer cell-conditioned media (Fig. 5d).

We next combined evaluation of lysosomal proteolysis with assessment of other elements of the lysosomal pathway in DCs from WT, *Ch25h*^ΔDC^, *Atf3*^ΔDC^, and *Ch25h*;^ΔDC^ *Atf3*^ΔDC^ mice. These cells displayed apparently comparable lysosomal parameters including levels of LAMP2 (Supplementary Fig. 5c), expression of genes involved in lysosomal biogenesis such as *Tfec*, *Tfeb*, *Tef3* and *Creg1* (Supplementary Fig. 5d), and lysosomal pH (Supplementary Fig. 5e). Knockout of either ATF3 or/and CH25H did not dramatically affect the lysosomal integrity as manifested by accumulation of FITC-conjugated dextran (Supplementary Fig. 5f). Furthermore, whereas these DCs exhibited a similar overall cholesterol content, levels of 25HC were increased upon ablation of *Atf3* and this increase was abrogated by co-deletion of *Ch25h* (Supplementary Fig. 5g).

However, the deletion of *Ch25h* in DCs notably increased lysosomal degradation of conjugated OVA, whereas deletion of *Atf3* (which suppresses CH25H expression) decreased this lysosomal activity. Importantly, the phenotype of *Atf3* ablation was reverted by concurrent deletion of *Ch25h* (Fig. 5e). To corroborate these conclusions, we employed an evaluation of beads-bound OVA degradation by assessing the amounts of OVA left on the beads after they were phagocytosed by DCs. Such analysis showed that ablation of ATF3 inhibited OVA degradation (Fig. 5f). Importantly, deletion of CH25H accelerated OVA degradation regardless of the status of ATF3, again supporting the notion that CH25H is a functionally important downstream target of ATF3.

Concurrently, we used a single organelle-based flow cytometry^45^ and evaluated these very phagosome-localized beads for their ability to acquire the LAMP2 lysosomal marker—a phenomenon indicative of the phago-lysosome fusion^46^. As seen from Fig. 5g, knockout of ATF3 in DCs decreased phagosomes fusion with lysosomes in these cells. Conversely, knockout of CH25H increased the extent of the phago-lysosome fusion and reversed the phenotype of ATF3 ablation. These results implicate the ability of the ATF3-CH25H pathway to control lipid membrane fusions in the regulation of the lysosomal degradation activity.

Taken together, our results thus far lend a hand to the following hypothesis: TMDF-activated ATF3 suppresses the expression of CH25H. Since CH25H restricts the lysosomal degradation of antigens, the net results of the ATF3-CH25H regulatory axis include an acceleration of antigen proteolysis and resulting poor cross-presentation of tumor antigens by the intratumoral DCs. Accordingly, this ATF3-CH25H axis limits activation of anti-tumor T cells and stimulates tumor growth.

### DC CH25H is an important factor of anti-tumor immunity and a target for STING agonist therapy

We next sought to examine the importance of downregulation of CH25H in DCs for anti-tumor immunity and tumor growth. To this end, we used *Ch25h*^ΔDC^ mice, which exhibited decreased levels of CH25H in CD11c^+^ cells (Supplementary Fig. 3h) yet did not differ in frequency of DCs or CD8^+^ T cells in the spleen (Supplementary Fig. 6a). We initially evaluated the ability of these mice to support growth of subcutaneous transplanted syngeneic MC38 colon adenocarcinoma tumors (chosen because of their high antigenic load^47^). Ablation of CH25H in DCs resulted in accelerated growth of MC38 tumors (Supplementary Fig. 6b), and decreased frequency and numbers of intratumoral CD8^+^ T cells (Supplementary Fig. 6c). Additional analyses of intratumoral CD8^+^ T cells from *Ch25h*^ΔDC^ mice (compared with those from *Ch25h*^f/f^ animals) indicated a decrease in expression of the activation markers CD69 and PD-1 (Supplementary Fig. 6c). Given that CH25H is already downregulated in the tumor microenvironment in WT mice (Fig. 2), these differences are likely underappreciated. Nevertheless, they indicate that downregulation of CH25H in DCs impedes the T cell-based anti-tumor immune responses and stimulates tumor growth.

**Fig. 6:**
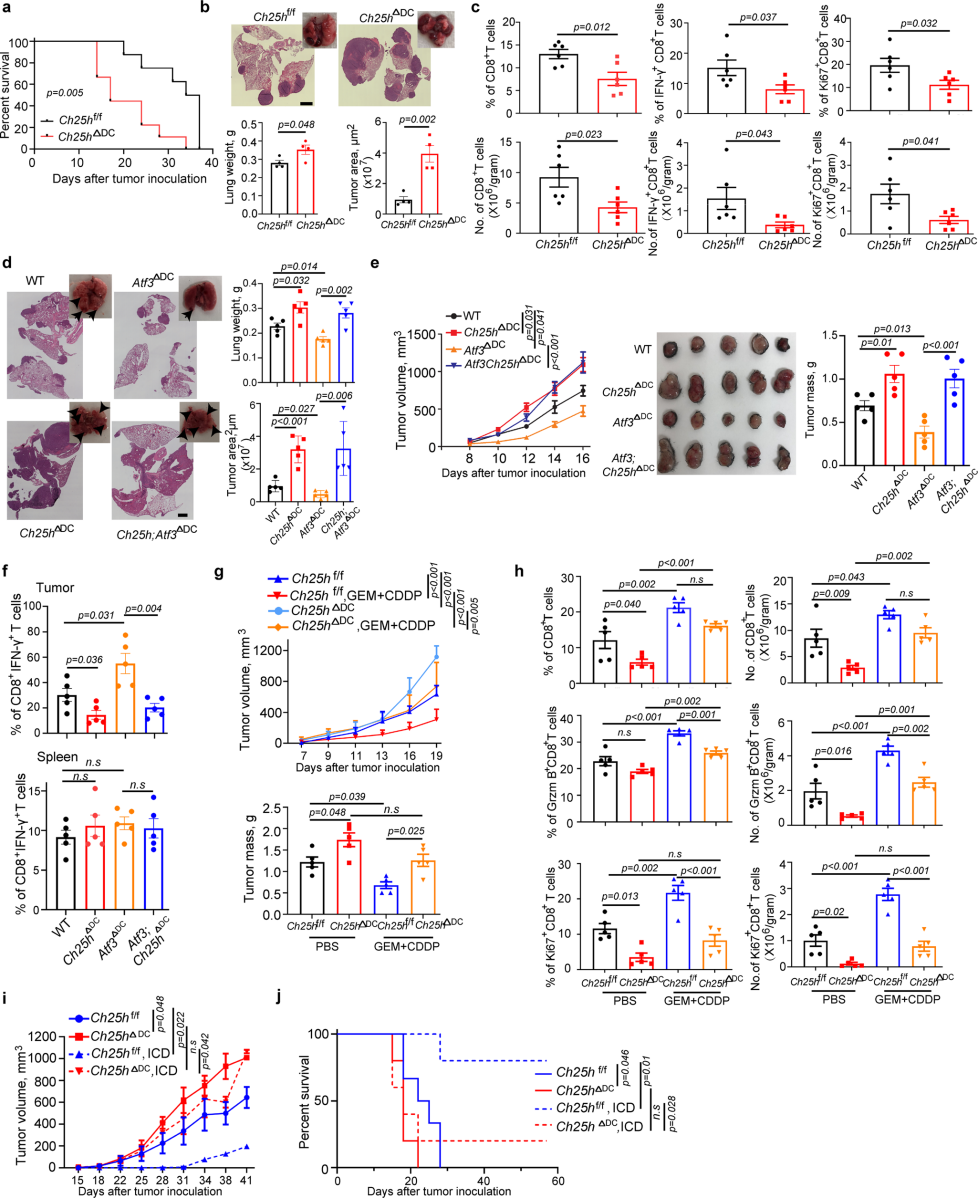
Downregulation of CH25H in DCs undermines the anti-tumor immunity and accelerates tumor growth. **a** Kaplan–Meier analysis of survival of LLC tumor-bearing mice after intravenous injection of 4 × 10^5^ LLC by log-rank test. *n* = 8 mice in *Ch25h*^f/f^ and *n* = 9 mice in *Ch25h*^ΔDC^ groups. **b** Tumor weight, representative lung images and the corresponding H&E-stained lung sections from *Ch25h*^f/f^ and *Ch25h*^ΔDC^ mice (*n* = 4 mice per group) 18 days after intravenous injection of 6 × 10^5^ LLC tumor cells. Scale bar:2 mm. Similar results were obtained from three independent experiments. **c** Flow-cytometric determination of the percentage and quantitative estimates of intratumoral CD8^+^ T cells in tumor lungs from *Ch25h*^f/f^ and *Ch25h*^ΔDC^ mice. *n* = 6 tumors per genotype. **d** Tumor weight, representative lung images and the corresponding H&E-stained lung sections from mice of indicated genotypes (*n* = 5 mice per group) 17 days after intravenous injection of 1 × 10^6^ LLC tumor cells. Scale bar: 1 mm. Similar results were obtained from three independent experiments. **e** Volume, appearance and mass of LLC tumors on Day 17 after s.c. inoculation (5 × 10^5^ cells/mouse) into mice of indicated genotypes. *n* = 5 mice per group. **f** Flow cytometry analysis of IFN-γ expression by CD8^+^ T cells isolated from LLC tumors or spleens of LLC-bearing mice from experiments described in Panel 6e. **g** Growth of LLC tumors in mice after s.c. injection of 6 × 10^5^ LLC cells into *Ch25h*^f/f^ and *Ch25h*^ΔDC^ mice. After 8 days of LLC inoculation, animals were treated with PBS or gemcitabine (GEM, 30 mg/kg, every 3 days, four times) plus cisplatin (CDDP, 3 mg/kg, every 6 days, twice). Tumor weight was measured at day 20 from tumor inoculation. *n* = 5 mice per group, data was shown in Mean ± SEM. **h** Flow-cytometric analysis of the percentage and quantitative estimates of intratumoral CD8^+^ T, Ki67^+^ and GzmB^+^ CD8^+^ T cells. *n* = 5 tumors in each group. **i** Volumes of LLC tumors in syngeneic mice that were either vaccinated with LLC cells undergoing immunogenetic cell death (ICD) or not vaccinated. Data were processed using the mixed-effect analysis of 2-way ANOVA with Tukey’s multiple comparisons test. *n* = 5 mice per group. **j** Kaplan–Meier analysis of survival of mice from experiment described in panel **i**. Data were processed using the Log-rank test. *n* = 5 mice per group. Data presented as mean ± SEM. Statistical analysis was performed using 1-way ANOVA with Tukey’s.l multiple-comparison test (D, E, F, G and H) test or 2-way ANOVA with multiple comparison (E, G and I) or 2-tailed Students’ *t*-test (B and C) or Kaplan-Meier survival analysis (A and J). n.s., not significant. Source data are provided as a Source Data file.

These conclusions were further corroborated in experiments involving the induction of lung tumors by intravenous inoculation of LLC cells. Compared to WT counterparts, *Ch25h*^ΔDC^ mice exhibited decreased survival (Fig. 6a) and increased pulmonary tumor load (Fig. 6b). In addition, lung tumors in these mice were characterized by lower frequency and number of CD8^+^ T cells. These intratumoral CD8^+^ T cells from *Ch25h*^ΔDC^ mice also displayed lesser levels of markers of activity (IFN-γ) and proliferation (Ki-67) (Fig. 6c). These results further support downregulation of CH25H in DCs as an important immunosuppressive mechanism contributing to tumor growth.

In a separate set of experiments, we aimed to examine the epistatic relationship between expression of ATF3 and CH25H in DCs and importance of this relationship for tumor growth. To this end, we inoculated LLC cells intravenously into WT, *Ch25h*^ΔDC^, *Atf3*^ΔDC^ or *Atf3;Ch25h*^ΔDC^ mice. Consistent with our earlier data, loss of ATF3 in DCs suppressed whereas ablation of CH25H stimulated tumor growth (Figs. 1d, e and 6a, b, d). Importantly, pulmonary tumor growth in *Atf3; Ch25h*^ΔDC^ mice mimicked the phenotypes of *Ch25h*^ΔDC^ animals (Fig. 6d) indicating that CH25H acts downstream of ATF3. Similar results were obtained in the subcutaneous tumor growth settings (Fig. 6e). Whereas DC numbers and CD86 expression were comparable in tumors from these animals (Supplementary Fig. 6d), the frequencies of active IFN-γ-expressing CD8^+^ T cells in the tumor (but not splenic) tissues were increased by ablation of ATF3 but decreased in response to the loss of CH25H in DCs regardless of the ATF3 status (Fig. 6f). In all, these results suggest that ATF3-driven downregulation of CH25H in DCs plays an important function in evading anti-tumor immunity and in stimulation of tumor growth.

Expression of tumor antigens encountered by the intratumoral DCs can be robustly stimulated by chemotherapy^48^. Here we sought to define the importance of CH25H expression in DCs under such conditions and chose a regimen that is relevant for human lung cancer treatments utilizing gemcitabine^49^ and cisplatin^50^. We examined the importance of CH25H expression in DCs by comparing subcutaneous growth of LLC tumors in WT and *Ch25h*^ΔDC^ animals that were administered a combination of these agents or a vehicle. Under these conditions, tumors in *Ch25h*^ΔDC^ mice grew faster and were somewhat less responsive to chemotherapy compared to control animals (Fig. 6g). Although we did not detect the effects of CH25H knockout on frequency, numbers or activation status of intratumoral DCs (Supplementary Fig. 6e), a notable decrease in infiltrating CD8^+^ T cells and their expression of Ki-67 or Granzyme B (Fig. 6h) was observed in tumors from *Ch25h*^ΔDC^ animals even after chemotherapy. These data support the notion that downregulation of CH25H in DCs attenuates their functions and anti-tumor immune responses under different conditions including a scenario wherein chemotherapy may increase the levels of tumor antigens.

To directly evaluate the importance of CH25H expression in DCs for the anti-tumor immune response we compared the ability of WT and *Ch25h*^ΔDC^ mice to detect antigens generated during the process of the immunogenic cell death (ICD) and develop long term protection against tumor challenge^51,52^. We immunized mice with LLC cells treated with oxaliplatin that induced ICD exemplified by cell death alongside release of calreticulin (Supplementary Fig. 6f) and then re-challenged them with naïve LLC cells inoculated into the opposite flank (Supplementary Fig. 6g). Under these conditions, knockout of CH25H in DCs led to a notable attenuation of the long-term anti-tumor immunity as seen from inadequate protection elicited by vaccine against tumor growth and death in *Ch25h*^ΔDC^ mice (Fig. 6I, j). Collectively, these results show that CH25H expression in DCs is required to mediate effective anti-tumor immunity generated by ICD inducing agents.

Given that loss of CH25H is associated with cancer immune evasion, it is plausible that agents preserving the levels of CH25H in DCs would exhibit immunostimulatory and anti-tumor properties. CH25H is an interferon-stimulated gene^53^; and these genes can be upregulated by activation of the stimulator of interferon genes (STING). Use of small molecules that act as the STING agonists capable of stimulating antigen presentation and increasing the immune response against cancer cells^54^ has emerged as an exciting and promising strategy for cancer therapy^55,56^.

**Fig. 7:**
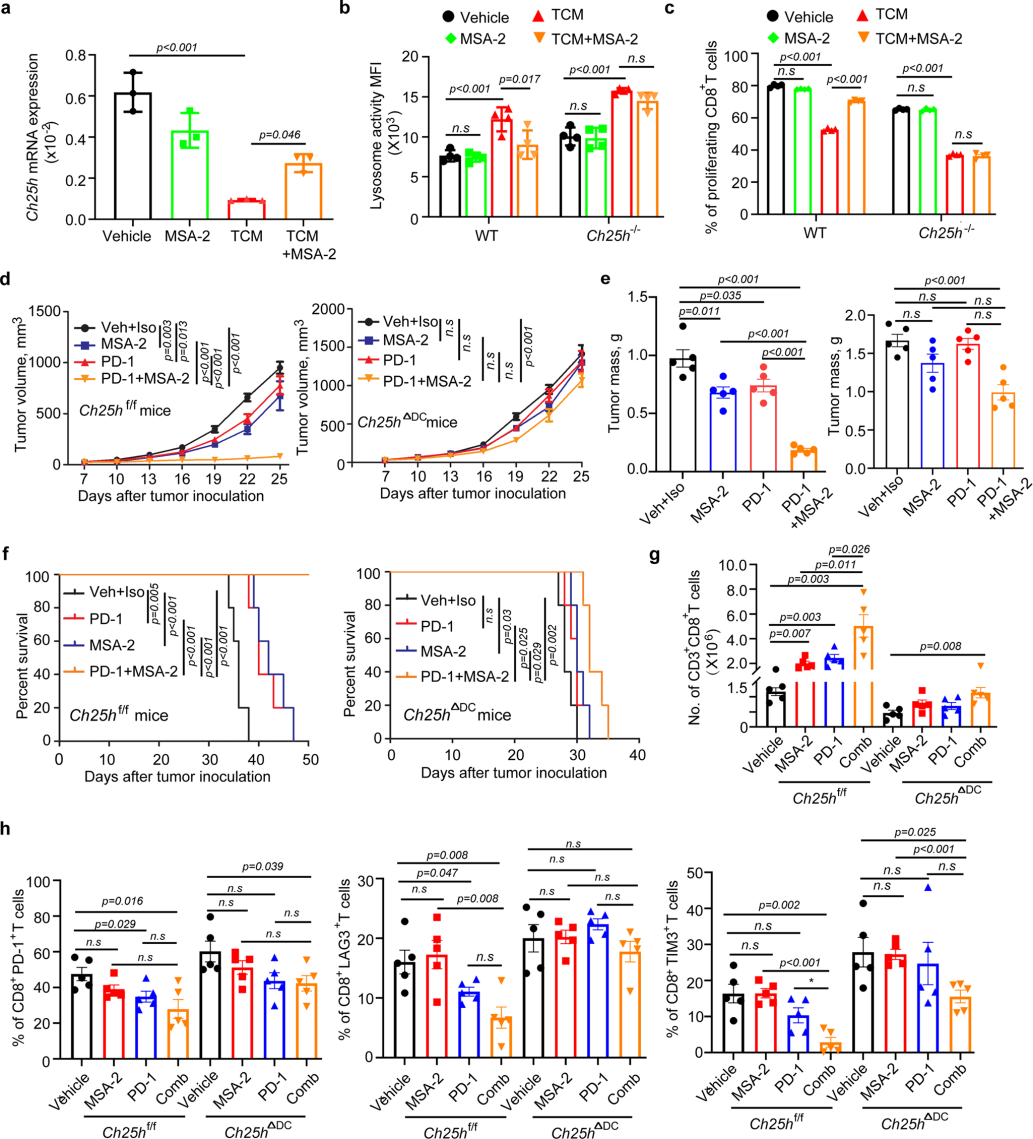
STING agonist acts to maintain CH25H expression in DCs to increase intratumoral immune infiltration and elicit anti-tumor therapeutic effects. **a** qPCR analysis of *Ch25h* mRNA in BMDCs pre-treated with MSA-2 (10 µM) for 30 min and LLC-conditioned media (TCM, 67%, *v/v*) for 2 h. *n* = 3 biologically independent samples. **b** Quantitative estimates of lysosome activity in WT or *Ch25h*^*−/−*^ BMDCs pretreated with LLC-conditioned media (TCM, 70% v/v) with or without pre-treatment of MSA-2 (10 µM, 2 h pretreatment) for 16 h. *n* = 4 biologically independent samples. **c** Antigen cross-presentation was assessed by proliferation of CFSE-labeled OT-I T cells co-cultured (10:1) with OVA-pulsed (200 μg/mL, 6 h) WT or *Ch25h*^*−/−*^ DCs for 72 h. DCs were pre-treated with medium conditioned by LLC cells (70%, v/v) for 20 h with or without the treatment of MSA-2 (10 µM) for 16 h before OVA pulse. *n* = 4 mice per group. **d** Volumes of LLC tumors grown in *Ch25h*^f/f^ (top panel) or *Ch25h*^∆DC^ (bottom panel) mice treated with Vehicle+Isotype control (anti-mouse IgG1 monoclonal antibody), anti-PD1 antibody (i.p, 5 mg/kg every 4 days), MSA-2 (orally, 60 mg/kg every 4 days) and combination. *n* = 5 mice per group. **e** Tumor mass of LLC tumors from experiment described in panel **d**. *n* = 5 mice per group. **f** Kaplan–Meier analysis of survival of LLC tumor-bearing mice described in panel **d**. *n* = 5 mice per group. **g** Flow cytometry analysis of number of CD3^+^CD8^+^ T cells in LLC tumors grown in *Ch25h*^f/f^ or *Ch25h*^∆DC^ mice with indicated treatment described in panel **d**. *n* = 5 mice per group. **h** Flow cytometry analysis of the percentage of CD8^+^ PD-1^+^, CD8^+^ LAG3^+^ and CD8^+^TIM3^+^ T cells in tumors from experiment described in panel **d**. *n* = 5 mice per group. Data presented as mean ± SEM. Statistical analysis was performed using 2-tailed Students’ *t*-test (A, B, C, E, G and H) or 2-way ANOVA with multiple comparison (D) or Kaplan-Meier survival analysis (F). n.s., not significant. Source data are provided as a Source Data file.

Contrary to our expectations, pre-treatment of WT DCs with the STING agonist MSA-2^55^ did not induce *Ch25h* expression under chosen conditions. However, MSA-2 treatment acted to partially prevent downregulation of *Ch25h* mRNA levels in response to LLC tumor cell conditioned media (Fig. 7a). Furthermore, in WT DCs, MSA-2 attenuated the increase in lysosomal degradation (Fig. 7b) and the suppression of antigen cross presentation (Fig. 7c, Supplementary Fig. 7a) elicited by tumor cell conditioned media. Importantly, the effects of MSA-2 were not evident in CH25H-deficient DCs (Fig. 7b, c) indicating that upregulation of CH25H is important for MSA-2-mediated restriction of lysosomal activity and stimulation of antigen cross presentation.

Consistent with previous report^55^, we noted a modest single agent activity of MSA-2 and a very robust anti-tumor effect of its combination with immune checkpoint inhibitor (anti-PD-1) against LLC tumors in WT mice (Fig. 7d–f). The therapeutic efficacy of MSA-2 was slightly attenuated in *Ch25h*^ΔDC^ mice. Growth of LLC tumors in these animals were also somewhat less responsive to monotherapy with anti-PD-1 antibody. Importantly, tumors in *Ch25h*^ΔDC^ mice were notably more resistant to a combination of STING agonist and immune checkpoint inhibitor as manifested by tumor volume and weight as well as animal survival (Fig. 7d–f).

Immunoprofiling of these tumors showed that MSA-2+anti-D-1 combination did not affect the numbers of CD4^+^ T cells or tumor-associated F4/80-positive macrophages (Supplementary Fig. 7b, c). However, tumors from WT mice treated with this combination exhibited increased numbers of the intratumoral CD8^+^ cytotoxic T lymphocytes (Fig. 7g) and greater levels of IFN-γ in these cells (Supplementary Fig. 7d). Accordingly, this regimen decreased exhaustion markers (including LAG3, PD-1 and TIM3) on the WT intratumoral CD8^+^ T cells (Fig. 7h). Importantly, all these changes were either attenuated or not observed at all in *Ch25h*^ΔDC^ mice. These results demonstrate the importance of CH25H in DC function for the generation of anti-tumor immune responses elicited by immunotherapies.

## Discussion

The anti-tumor functions of DCs are undermined by immune suppressive tumor microenvironment (reviewed in ref. 6). Our current studies implicate a mechanism of DCs inactivation that involves upregulating ATF3 and downregulating CH25H. We demonstrate that factors present in the tumor microenvironment (such as PGE_2_, VEGF and likely others) activate this ATF3-CH25H regulatory axis to increase the lysosomal activity in the intratumoral DCs leading to degradation and poor cross-presentation of tumor antigens. Partial loss of CH25H expression occurs in human intratumoral APC and is associated with lung cancer growth and progression. A decrease of CH25H in DCs stimulates lysosomal activity and suppresses antigen cross presentation leading to attenuated anti-tumor immunity and accelerated growth of lung tumors. In addition to previously described mechanisms including NF-κB-driven inhibition of DC maturation^14^, suppression of their migration^11^ and viability^12^, our results place the dysregulation of the ATF3-CH25H axis as an important pathway, through which tumors inactivate DCs and evade the immune system.

These findings suggest an important function for ATF3 and CH25H as an important regulator of lysosomal degradation in the context of tumor growth. A critically important activity of fine-tuning lysosomal activity in the regulation of antigen cross presentation has been well established^19–21^. Maturation and function of lysosomes is affected by altered membranes lipid components and the ability of membranes to fuse^43,44^. Importantly, lipid membrane fusion is suppressed by the CH25H product, 25HC^31,32^, which appears to act as a negative regulator of lysosomal activity (Fig. 4). Importantly, demonstrated stimulating effects of TMDF on lysosomal activity show an unexpected mechanism by which dysregulation of the lysosomal function can contribute to immune evasion and tumor growth and progression. However, our results do not rule out a putative importance of the ATF3-CH25H pathway in the regulation of antigen cross presentation via non-vacuolar yet proteasome and/or transporter associated with antigen processing (TAP)-dependent mechanisms.

Our results cannot exclude the importance of ATF3 and CH25H in the control of key genes that encode lysosomal cathepsins and their regulators. Of note, lysosomes are central regulators of degradation of oxidized lipids, which, in turn, can dysregulate the lysosomal function^57,58^. Accordingly, elevated levels of oxidized triglycerides, fatty acids and cholesterol ethers in DCs have been indeed associated with defects in cross-presentation^59–61^. The translational importance of these reports along with our current findings is reflected in emerging efforts to improve the lysosomal inhibitors for cancer treatment^62,63^.

Data presented here are consistent with increased anti-tumor immunity and restricted tumor growth observed in knockout mice lacking the expression of palmitoyl-protein thioesterase 1, which normally acts to increase lysosomal activity and to promote antigen degradation in DCs^64^. Conversely, we demonstrate that tumor microenvironment-associated factors activate ATF3 and downregulate CH25H and its product 25HC thereby alleviating the negative regulation of the lysosomal activity. In theory, the latter can have grave consequences far beyond the impediment of the antigen cross presentation, which was a focus of our current study. On one hand we did not observe effects of CH25H ablation on cell surface expression of MHC-I, which can be a lysosomal substrate^65^. On another hand, it remains plausible that a hyper-stimulated lysosomal activity may increase the rate of proteolytic turnover and induce downregulation of many types of cell surface proteins that are normally degraded in the lysosomes.

Among those could be the CCR5 and CCR8 chemokine receptors that recruit DCs into melanoma tumor microenvironment in response to CCL4 chemokine^8^. However, we did not detect consistent and reproducible changes in the frequencies of these DCs in tumors (Supplementary Fig. 6d). Likewise, ablation of CH25H also did not downregulate MHC-I levels (Fig. 3) suggesting that additional regulators help to discriminate the fate of diverse proteins that could become lysosomal substrates in DCs. Further studies focused on other physiological and pathological consequences of alterations in activities of lysosomes and their endocytotic and autophagy targets that are affected by the dysregulation of the ATF3-CH25H pathway are warranted.

Although presented here data indirectly suggest that targeting ATF3 in DCs for inactivation may yield clinical benefits against lung cancer, two important considerations might complicate potential therapeutic approaches. First, ATF3 may exhibit not only pro-tumorigenic but also tumor suppressive properties^23^. Second, it might be difficult to specifically disrupt the ATF3-CH25H pathway in DCs without evoking potentially harmful effects of augmenting macrophage foam cell formation atherosclerosis otherwise suppressed by ATF3 and promoted by CH25H^37^. Perhaps a more rationale direction towards reactivation of the intratumoral DCs would be the development of pharmacologic agents that mimic effects of 25HC in antigen cross presentation but not in atherosclerotic processes.

CH25H is an interferon-inducible gene^53^, and type I interferon pathway has been implicated in efficacy of chemotherapy against cancers^66,67^. Furthermore, DCs act as critical cellular mediators of ICD^68^. Our current data shows that expression of CH25H DCs impacts the activity of STING agonist and is required for the induction of effective anti-tumor immunity secondary to ICD. Beyond the mechanistic salience, this observation has further implications for the treatment of lung cancer patients. Numerous ICD-inducing chemotherapeutics have been investigated for NSCLC patients and hold promise for the treatment of this disease^69^. In addition, the prospects of activating STING for therapeutic purposes is very exciting^55,56^. Future clinical studies will further establish whether the status of CH25H and lysosomal activities in DCs may help with stratifying which patients will respond favorably to the regimens of ICD inducers and STING agonists.

## Methods

### Study approvals

All research complies with all relevant ethical regulation. Studies involving collection and use of cells from human patients adhered to the US Common rule and were also compliant with the declaration of Helsinki and the Belmont report. A total of 18 patients with Stage I–III lung cancer, who were scheduled for surgical resection, were consented for tissue collection. A portion of their tumor and non-cancerous adjacent lung tissue was used for research purposes at the Hospital of the University of Pennsylvania and The Philadelphia Veterans Affairs Medical Center after obtaining consents that had been approved by their respective Institutional Review Boards. Detailed characteristics of the patients can be found in refs. 5, 41. All animal experiments were approved by the Institutional Animal Care and Use Committee (IACUC protocol 803995) of the University of Pennsylvania and were carried out in accordance with the IACUC guidelines.

### Preparation of a single-cell suspension from human tumor and adjacent lung tissue

Fresh, surgically resected lung tumors and adjacent lung tissue were processed within 20 min of removal from the patient. We used an optimized disaggregation method for human lung tumors that preserves the phenotype and function of the immune cells^70^. Briefly, under sterile conditions, all areas of tissue necrosis were trimmed away and discarded. The tumor and adjacent uninvolved lung tissue were sliced into 1–2 mm^3^ pieces with micro-dissecting scissors equipped with tungsten carbide insert blades (Biomedical Research Instruments, Inc. Silver Spring, MD). For enzymatic digestion, the minced pieces of tissue were then incubated in a shaking incubator for 45 min at 37 °C in serum-free L-15 Leibovitz media (HyClone) containing enzymes at low concentrations (see specifics above) and 1% Penicillin-Streptomycin (Life Technologies, Carlsbad, CA). L-15 Leibovitz media was formulated for use in carbon dioxide-free systems. After 45 min, any visible tumor pieces were vigorously pipetted against the side of a 50 mL tube to enhance disaggregation and then further incubated for 30–50 min under the same conditions. Larger pieces of tumor tissue were permitted to settle to the bottom of the tube and the supernatant was passed through a 70 μM nylon cell strainer (BD Falcon). The remaining pieces of undigested tissue in the tube underwent further pipetting before being passed through the same cell strainer. Typically, <5% of the tissue (consisting of chiefly non-cellular connective tissue) remained on the cell strainer. After filtration, the red blood cells were lysed using 1x Red Blood Cell (RBC) Lysis Buffer (Santa Cruz, Dallas, TX). The remaining cells were washed twice in RPMI supplemented with 2% FBS and re-suspended in the cell culture media. Cell viability, as determined by trypan blue exclusion or Fixable Viability Dye eFluor 450 staining, was typically >90%. If the viability of cells was <80%, dead cells were eliminated using a “dead cell removal kit” (Miltenyi Biotec Inc., Germany).

### Isolation of human macrophage/monocyte lineage cells from human lung tumors

All patients with LLC (including 14 males and 6 females) involved in this study were 51–84 old with informed consent and provided with appropriate compensation. We used a combination of our tumor digestion protocol and microbeads to isolate cells of monocyte/macrophage lineage from digested tumor, distant lung tissue and peripheral blood as previously described^5^. Macrophage/monocyte lineage cells (MMLC) were isolated from single cell suspensions using positive selection of CD14^+^ cells with microbeads (Miltenyi Biotec Inc.) with 95% purity. To account for any possible effect of tissue digestion enzymes on MMLC function, peripheral blood monocytes were processed in a similar manner. Isolated CD14^+^ cells demonstrated a high cell viability with minimal cleavage of myeloid cell markers^5,70^. These samples were analyzed by reverse transcription quantitative polymerase chain reaction (qRT-PCR). Investigators conducting the qPCR were blinded as to whether the samples were derived from which patient.

### Animal studies

All mice used in experiments were 6–8 weeks old males; these mice were fed regular chow and water *ad libitum*. Mice were maintained under specific-pathogen-free conditions in accordance with American Association for Laboratory Animal Science guidelines. WT (C57BL/6 J, Stock No. 000664), *Ch25h*^−/−^ (B6.129S6-*Ch25h*^tm1Rus^/J, Stock No. 016263), OT-I (C57BL/6-Tg(TcraTcrb)1100Mjb/J, Stock No. 003831) and *CD11c-cre* (B6.Cg-Tg(Itgax-cre)1-1Reiz/J, Stock No. 008068) mice were purchased from The Jackson Laboratory. The conditional *Ch25h* allele (*Ch25h*^f/f^) was created as previously described^71^. These mice are in the process of being donated to the Jackson Laboratory (future Stock No. 037647; C57BL/6-Ch25h^tm1.1Syfu^/J). *Atf3* flox allele (*Atf3*^f/f^) were described previously^27^. All mice were in C57BL/6 J background. *CD11c*-cre mice were crossed with *Ch25h*^f/f^ mice or *Atf3*^f/f^ mice to generate *CD11c-cre; Ch25h*^f/f^ mice (*Ch25h*^ΔDC^), *CD11c-cre;Atf3*^f/f^ mice (*Atf3*^ΔDC^), or *CD11c; Atf3*^f/f^ *Ch25h*^f/f^ mice litter-mates. The genotyping PCR primers are provided in Supplementary Table 1.

### Cell culture

Mouse colon adenocarcinoma MC38 cells and Lewis lung carcinoma (LLC) cells were purchased form ATCC and maintained in DMEM (Gibco) supplemented with 10% FBS (HyClone) and 100 U/ml Penicillin-Streptomycin (Gibco).

Mouse splenocytes and bone marrow-derived cells (BMDCs) were isolated as previously described^72^ and maintained in 10% FBS containing RPMI-1640 medium (Sigma). For CD11c^+^ myeloid cells, BMDCs were cultured in differentiation medium (RPMI-1640 medium containing mouse recombinant granulocyte-macrophage colony-stimulating factor (10 ng/ml, Invitrogen)) and mouse recombinant Interleukin-4 (10 ng/ml, Invitrogen) for 5–7 days, the cells were harvested and purified by EasySep Mouse CD11c Positive Selection Kit II (StemCell, #18780). All cells were incubated at 37 °C in a 5% CO_2_ humidified environment.

### Tumor models, treatments and analysis

For the syngeneic subcutaneous tumor model, all age-matched mice (6–8-week-old) were shaved at the flank. LLC or MC38 tumor cells were injected into the shaved, right flank of indicated mice. Tumor volume was measured by caliper and calculated using the formula: width^2^ × length/2. For the pulmonary tumor growth, LLC cells were injected into the tail vein. Mice were euthanized at the indicated time and the lungs were collected followed by either immunohistology analysis or flow cytometry. Quantification of lung tumor lesions was carried out as previously described^33^.

The maximal tumor size permitted by IACUC was <20 mm in diameter; this maximal tumor size was not exceeded in described experiments. Mice were euthanized when >20% of body weight was lost or when tumor volume approached ~1500 mm^3^, or when tumors became ulcerated.

In the experiment involving Gemcitabine (GEM, Selleckchem, #s1149) and Cisplatin (CDDP, Selleckchem, #s1166), age- and sex-matched *Ch25h*^f/f^ mice and *Ch25h*^ΔDC^ mice (*n* = 10 for each genotype) were inoculated subcutaneously with LLC cells (6 × 10^5^ per mouse). Chemotherapy started at Day 8 when tumors reaching to ~100 mm^3^. Before treatment, *Ch25h*^f/f^ mice and *Ch25h*^ΔDC^ mice were randomized into two groups (*n* = 5 per group). One group receiving PBS which served as the control group. The other groups were injected intraperitoneally with GEM at 30 mg/kg on Day 8, 11, 14 and 17 plus CDDP at 3 mg/kg on Day 11 and 17. Tumor size was measured every other 2 days. On day 20, tumor and spleen were excised, and single cell suspension was prepared for further analyses.

In the experiment evolving anti-PD-1 and MSA-2 combination, age- and sex-matched Ch25h^f/f^ mice and Ch25h^ΔDC^ mice (*n* = 20 for each genotype) were inoculated subcutaneously with LLC cells (5 × 10^5^ per mouse). For each genotype, the syngeneic tumor-bearing mice were randomly divided into four groups. They were treated with vehicle, PD-1 mAb (i.p., 5 mg/kg, on day 4, 8, 12 and 16, BIO-XCELL), MSA-2 (orally, 40 mg/kg, on day 4, 8, 12 and 16, MedKoo Biosciences) or PD-1 mAb combined with MSA-2 at indicated doses and dosing intervals. Tumor size was measured every other 2 days. For the immunoprofiling analyses, tumor tissues were dissected and digested with 1 mg/mL Collagenase D (Roche, 11088882001) with 100 μg/mL DNase I (Roche, 10104159001) in RPMI medium with 2% FBS for 1 h with continuous agitation at 37 °C. The digestion mixture was passed through a 70-μm cell strainer to prepare a single-cell suspension and washed with PBS supplemented with 2 mM EDTA and 1% FBS. The single cells were stained with antibodies listed in Supplementary Table 2.

### Isolation of cells from mouse tumors and tissues

Tumor tissues were dissected and digested in RPMI medium containing 2% FBS, 1 mg/mL Collagenase D (Roche, #11088882001) and 100 μg/mL DNase I (Roche, #10104159001) for 1 h with continuous agitation at 37 °C. Lung tissues were digested in PBS containing 2 mg/mL Collagenase II (MP Biomedicals), 1 mg/mL Collagenase D and 100 μg/mL DNase I for 1 h. The digestion was filtered through a 70-μm cell strainer to prepare a single-cell suspension. Red blood cells were lysed in lysing buffer (BD Bioscience) and the remaining cells washed and resuspended in PBS.

Lung draining lymph node (LN) was isolated following the methods as previously described^73^. LN and spleen were excised, mashed, and filtered through a 70 µm filter. Red blood cells were lysed in RBC lysis buffer and the remaining cells were resuspended in cold PBS.

MSCV-Ch25h-IRES-Thy1.1 vectors for retroviral expression of WT CH25H or catalytically inactive mutant (H242, 243Q) of CH25H were a generous gift of Dr. J.G. Cyster (UCSF). Retroviruses for transduction were prepared by transfecting Phoenix packaging cells. DCs were isolated from spleens of *Ch25h*^−/−^ mice and transduced with retroviruses that expressed wild type or mutant CH25H for 48 h. After that, naïve or transduced *Ch25h*^−/−^ DCs were pulsed with soluble OVA (200 μg/ml) for 6 h followed by co-culture (10:1) with OT-I T cells for 72 h. T cell proliferation was tested through analyzing CFSE dilution. For lysosome degradation/activity assay, indicated DCs were incubated with DQ ovalbumin (10 µg/mL, Invitrogen, #D12053) for 30 min followed by flow cytometry analysis.

### Flow cytometry analysis

All gating strategy information is depicted in the Supplementary Fig. 8. Single cell suspensions from the indicated tissues were prepared as described above. Cells were resuspended in staining buffer (PBS, 1% FBS, 1 mM EDTA), followed by blocking of the Fc receptor and staining with the indicated antibodies for 30 min on ice in the dark. All antibodies used for flow cytometry (listed in Supplementary Table 2) were diluted as 1:200. For intracellular staining, cells were stained according to recommendations of the manufacturer of the eBioscience Foxp3 / Transcription Factor Staining Buffer Set (Cat No: 00-5523-00). Cells were washed and resuspended in staining buffer or PBS and either analyzed on LSRFortessa flow cytometry (BD Biosciences) or Canto flow cytometry (BD Bioscience). Data were analyzed with FlowJo V10.7.1(BD) software.

### Quantitative PCR (qPCR)

Total RNA was extracted using TRIzol (Invitrogen) and cDNA was prepared using the High-Capacity cDNA Reverse Transcription Kit (Life Technologies). Real-time qPCR was performed using SYBR reagent (Applied Biosystems). The fold changes of gene transcript levels were normalized by the housekeeping gene using the 2^-ΔΔCq^ method. The primer sequences are listed in Supplementary Table 1.

### Immunoblotting and ELISA measurements of cholesterol and 25HC

Human monocytes were lysed in RIPA buffer to extract the whole cell lysate or lysed following the two-step membrane-bound protein isolation protocol^74^ for CH25H detection. The lysates were subjected to western blot analysis. Briefly, blotted membrane was blocked with 5% BSA (Sigma) containing PBS for 1 h, incubated with primary antibodies (Abs) overnight, and incubated with secondary Abs in blocking buffer. β-actin (Sigma, clone AC-15) and Na, K-ATPase 1 protein (CST, #3010 S) was served as loading control, respectively. ATF3 and CH25H were detected using anti-ATF3 Ab (CST, clone: D2Y5W) and anti-CH25H Ab (Invitrogen, #PA5-72329), respectively, and IRDye 680RD donkey anti-rabbit secondary antibody (LI-COR Bioscience). Results were analyzed using a LI-COR CL Odyssey imager.

For analyses of cholesterol and 25HC levels, DCs were isolated from spleen of indicated mice and cell lysate was made using lysis buffer from Promega Cholesterol/Cholesterol Ester-Glo assay kit (#J3190) or by multiple thaw-freeze cycles. ELISA-based analyses of the levels of cholesterol or 25HC was carried out using Cholesterol/Cholesterol Ester-Glo assay ELISA kit (Promega, #J3190) or Mouse 25-hydroxycholesterol ELISA kit (MyBioSource, # MBS7256104) according to the manufacturers’ recommendations.

### RNA microarray

Bone marrow-derived macrophages (BMDMs) were isolated as described in ref. 27. Briefly, femurs and tibia from WT or *Atf3*^−/−^ mice were removed and cleaned. The tips of the bones were cut off and bones were flushed with PBS. The bone marrow was filtered through a 40 µm filter, pelleted, and RBC lysis was performed with BD Pharm Lyse Buffer (BD Bioscience). The remaining cells were cultured in complete DMEM with 20% L929-conditioned media for 7 days. BMDMs were then collected, and their RNA isolated using TRIzol. RNA was then used in an Agilent-014868 Whole Mouse Genome Microarray performed according to the manufacturer’s instructions.

### Antigen cross-presentation assay

The CD11c^+^ myeloid cells were treated with PGE_2_ (10 ng/mL, Sigma, #P0409), mouse VEGF (20 ng/mL, Sigma, #SRP4364), or LLC tumor conditioned media at indicated ratio for 24 h. In the last 4 h of treatment, DC661 (5 µM, Selleckchem, #s8808), 25HC (50 nM, Sigma, H1015), or MSA-2 (10 µM, MedKoo Biosciences, # HY-136927) was added into the media, respectively. The cells were then washed with fresh RPMI-1640 medium and pulsed with 50, 100 or 200 µg/ml OVA protein for 6 or 18 h. After that, cells were washed twice with PBS. Meanwhile, naïve CD8^+^ T cells were isolated and purified from spleen of OT-I mouse using EasySep Mouse Naïve CD8^+^ T Cell negative isolation kit (Stemcell, #19858 A). OT-I T cells were labeled with 1 µM CFSE (BioLegend, #423801) by incubation at 37 °C in the dark for 20 min. The CD11c^+^ cells were then co-cultured with CFSE-labeled OT-I cells at the indicated ratio and time. Cells were maintained in 24-well plates in 1 ml RPMI-1640 medium for 72 h. CFSE-labeled OT-I cells only were kept as negative proliferation control. Cells were collected and stained with anti-CD45, anti-CD3, anti-CD8 antibodies, followed by subsequent gating on CD8^+^ T cells. Dilutions in CFSE content of CD45^+^CD3^+^CD8^+^ OT-I cells were determined as a measure of T-cell proliferation.

For experiments using beads-bound OVA (bbOVA), we prepared these beads as previously described^45^. Briefly, 100 μl Polybeads Amino Microspheres beads (Polyscience, # 17145-5) were washed with PBS and treated with 8% glutaraldehyde for 6 h at room temperature. After that, beads were washed twice with PBS and incubated with OVA whole protein (0.5 mg/ml, Worthington, # LS003049) and bovine serum albumin at different ratios at 4 °C overnight. Finally, beads were washed and blocked with 0.5 M Glycine for 30 min followed by washing with cold PBS twice.

DCs were treated with different TDFs (with or without DC661 or 25HC as indicated) and incubated with OVA beads covered with different ratio of BSA and OVA whole protein (100% bbOVA, OVA 10 μg/μl alone; 50% bbOVA, OVA 5 μg/μl and BSA 5 μg/μl; 25% bbOVA, OVA 2.5 μg/μl and BSA 7.5 μg/μl). After 1 h incubation, DCs were washed with PBS three times and co-cultured with CFSE labeled OT-I T cells for 72 h. For tracking T cells proliferation, dilution of CFSE on CD8^+^ population was analyzed using BD Bioscience FacsCanto II Flow cytometer.

### Lysosome integrity, biogenesis and pH analyses

For lysosome integrity and pH assessments, DCs were isolated from spleen of genetically different mice and seeded into poly-D-lysine pre-coated dish. FITC-Dextran (0.1 μg/μl, Sigma, #FD10S) were added into cells and incubated for 12 h. After removing medium, cells were washed with cold PBS for 3 times and kept in fresh medium for 4 h. After that, part of the cells was subjected to image capture of FITC-Dextran distribution using confocal microscopy (100X) and analyzed by Image J 1.5.3/Image studio Lite V5.2 Another part of cell was subjected to flow cytometry to assess the pH by FITC intensity using two filter flow cytometry using FL1 channel (SP 550 nm filter and a BP 525 + 40 nm filter) and FL2 channel (SP 595 nm filter and a BP 575 nm filter). The FL1/FL2 parameters ratio are characteristic of pH levels.

For assessment of the lysosome biogenesis, DCs were isolated, washed with PBS, pelleted and incubated with 700 μl of 4% paraformaldehyde for 10 min at 37 °C followed by centrifugation at 500 g for 5 min. Cells were then washed with PBS and then incubated with 700 μl 100% cold methanol for 5 min at −20 °C followed by centrifugation at 500 g for 5 min. Cells were washed again with PBS and then incubated with 700 μl 0.1% Triton-100 for 15 min, pelleted again (500 g for 5 min), washed and blocked with 2% BSA at ambient temperature for 60 min. Finally, cells were incubated with CD107 Monoclonal Antibody (0.05 μg/μl, Invitrogen, #11-1072-81) for 1 h followed by flow cytometry analysis.

### Lysosomal degradation assay

Lysosomal Intracellular Activity Assay Kit (BioVision, #K448) was initially used for assessment of lysosomal cleavage of a self-quenching substrate. Subsequently, most of experiments used DQ ovalbumin (10 µg/mL, Invitrogen, #D12053), which was added into each cell suspension (1 mL per sample) and incubate at 37 °C for 30 min. Cells were collected and washed with PBS once and then stained with flow antibodies of DC markers (anti-CD45, anti-CD11c and anti-MHC II Abs). Lysosome activity was detected by FACS using 488 nm excitation laser (515/20 Blue channel of LSRFortesssa flow cytometer).

### Flow organellocytometry

The extent of the phago-lysosome fusion was evaluated by a single organelle-based flow cytometry^45^ that enabled analysis of bead-bound OVA degradation and acquisition of the LAMP-2 lysosomal marker using as previously described^46^. Briefly, for degradation, DCs were incubated with bbOVA (cell to beads ratio of 1:3) at 37 °C for 10 min. After that, 1 ml of cold PBS was added to stop phagocytosis and the cell-beads mixture was gently overlaid over cold fetal bovine serum and pelleted (150 g for 5 min); these steps were repeated twice. After that, cells were resuspended in the complete media and lysed at 0, 1 h and 2 h time points. The isolated beads were stained with poly anti-OVA antibody (Sigma, # C6543, 5000X) and goat anti-rabbit secondary antibody (Invitrogen, #A-11008, 500X). Samples were analyzed with BD Bioscience FacsCanto II Flow cytometer.

For assessment of the phagosomal acquisition of lysosomal marker, DCs were incubated with beads (cells to beads ratio of 1:5) at the same conditions prior to stopping phagocytosis with cold fetal bovine serum. Cells were then pelleted and mechanically disrupted using the 22-gauge needle. Beads were pelleted (500 g for 4 min), washed with PBS and stained with anti-LAMP-2 antibody (1:50, BD Bioscience, #550292) and goat anti-rat secondary antibody (1:500, Invitrogen, # A-11007). Samples were analyzed using BD Bioscience FacsCanto II Flow cytometer.

### Statistics and reproducibility

Statistical analysis was performed using GraphPad Prism 8 software (GraphPad). Unless indicated otherwise in the figure legends, results are shown as means ± SEM. Two-tailed unpaired Student’s t-test was used for the comparison between two groups. One-way analysis of variance (ANOVA) or two-way ANOVA followed by the Sidak’s, Dunnett’s or Tukey’s multiple comparisons test was used for the multiple comparisons. Repeated-measures two-way ANOVA (mixed model) followed by the Tukey’s multiple comparisons test was used for comparing the tumor growth curves. Log-rank test was used to compare between Kaplan-Meier curves. *P*-value < 0.05 was considered as significant.

### Reporting summary

Further information on research design is available in the Nature Research Reporting Summary linked to this article.

## Supplementary information

Supplementary Information

Reporting Summary

## Data Availability

Gene expression microarray data have been previously reported^28^ and deposited in the NCBI Gene Expression Omnibus (GEO) repository under accession number GSE164611. All other data supporting the findings of this study are available within the paper and its supplementary information files. Source data are provided with this paper.

## Source data

Source Data
