# Supplementary material for: Tumor factors stimulate lysosomal degradation of tumor antigens and undermine their cross-presentation in lung cancer
Source: Nat Commun. 2022 Nov 4;13:6623. doi: 10.1038/s41467-022-34428-w (PMC9636202; doi:10.1038/s41467-022-34428-w)
Supplement: Supplementary file 1 — Supplementary Information [file 41467_2022_34428_MOESM1_ESM.pdf]

Supplementary Fig.1

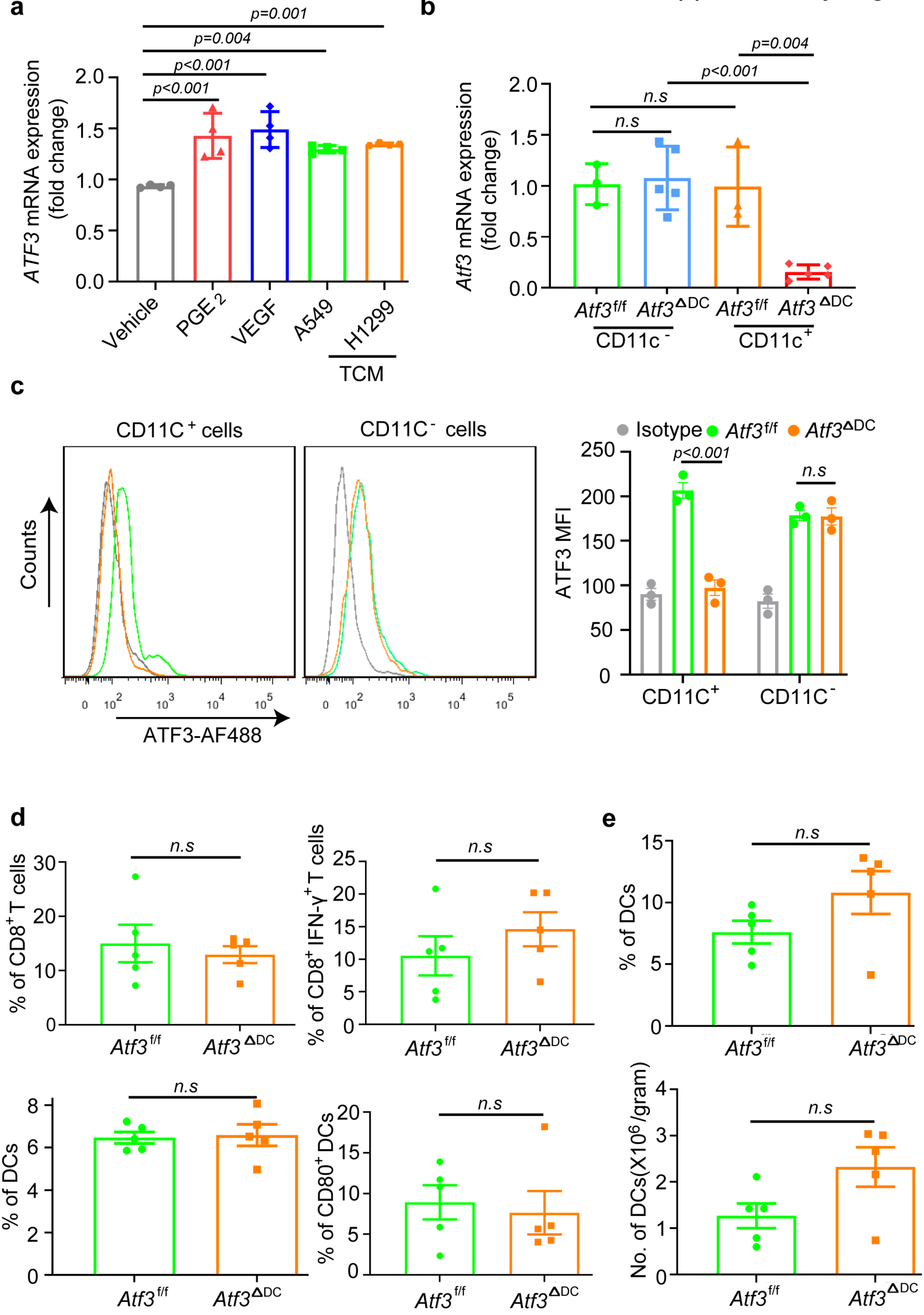

Supplementary Figure 1. Tumor microenvironment factors-induced ATF3 in DCs promotes tumor growth.

a. qPCR analysis of *ATF3* mRNA in human monocytes treated with vehicle, PGE_2 (10 ng/mL), VEGF (20 ng/mL), or TCM from A549 or H1299 cells (75%, v/v) for 2 hr. *n* = 4 biologically independent samples.

b. qPCR analysis of *ATF3* mRNA levels in $CD11c^+$ and $CD11c^-$ splenic myeloid cells from $Atf3^{f/f}$ and $Atf3^{\Delta DC}$ mice. *n* = 3 mice for $Atf3^{f/f}$ group and n=5 mice for $Atf3^{\Delta DC}$ group.

c. Flow cytometry analysis of ATF3 expression in $CD11c^+$ and $CD11c^-$ cells isolated from $Atf3^{f/f}$ and $Atf3^{\Delta DC}$ mice. n=3 mice per group.

d. Flow cytometry analysis of percentage of $CD8^+$ T cells or DCs and their corresponding absolute cell number in spleens of $Atf3^{f/f}$ and $Atf3^{\Delta DC}$ mice. n=5 mice per group.

e. Flow-cytometry determination of the percentage and quantitative estimates of intratumoral DCs from the experiment described in panel 1f. *n* = 5 tumors in each group.

Data presented as mean±SEM. Statistical analysis was performed using 2-tailed Students' t test (a, c, d and e) or 1-way ANOVA with Tukey's multiple comparison test (b). n.s., no significant. Source data are provided as a Source Data file.

Supplementary Fig.2

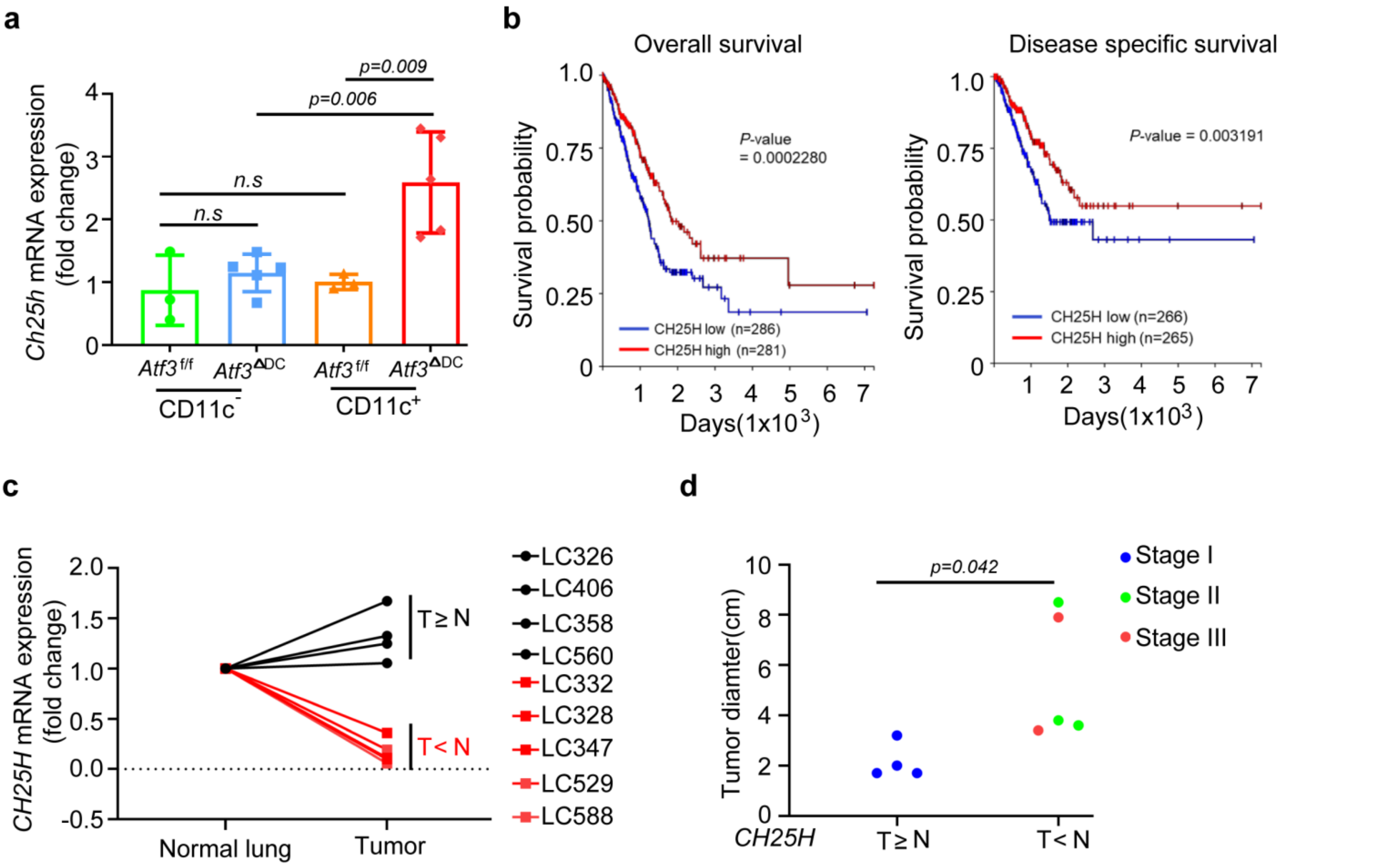

Supplementary Figure 2. Downregulation of CH25H in human and mouse lung tumors.

a. qPCR analysis of *Ch25h* mRNA levels in $CD11c^{+}$ and $CD11c^{-}$ splenic myeloid cells from *Atf3*$^{f/f}$ and *Atf3*$^{\Delta DC}$ mice. *n* = 3 mice for *Atf3*$^{f/f}$ group and n=5 mice for *Atf3*$^{\Delta DC}$ group. Data presented as mean±SEM; statistical analysis was performed using 1-way ANOVA with Tukey’s multiple comparison test.

b. Overall and disease specific survival among patients with lung adenocarcinoma with high or low expression of *CH25H* from TCGA Lung Adenocarcinoma (LUAD) database.

c. Comparisons of *CH25H* expression in $CD14^{+}$ monocytes from paired primary lung tumors and distant lung tissues from the same NSCLC patients. The samples were divided into two separate subsets based on the expression level of *CH25H*: a comparable *CH25H* levels within tumor and normal lungs (T≥N) and a notably lower level of *CH25H* in intratumoral $CD14^{+}$ monocytes compared to cells isolated from normal lungs (T<N).

d. Comparisons of tumor size and stage of human NSCLC cancer patients described in panel S2c. n=4 for stage I patients, n=3 for stage II patients and n=2 for stage III patients. Statistical analysis was performed using 2-tailed Students’ t test.

Source data are provided as a Source Data file.

Supplementary Fig.3

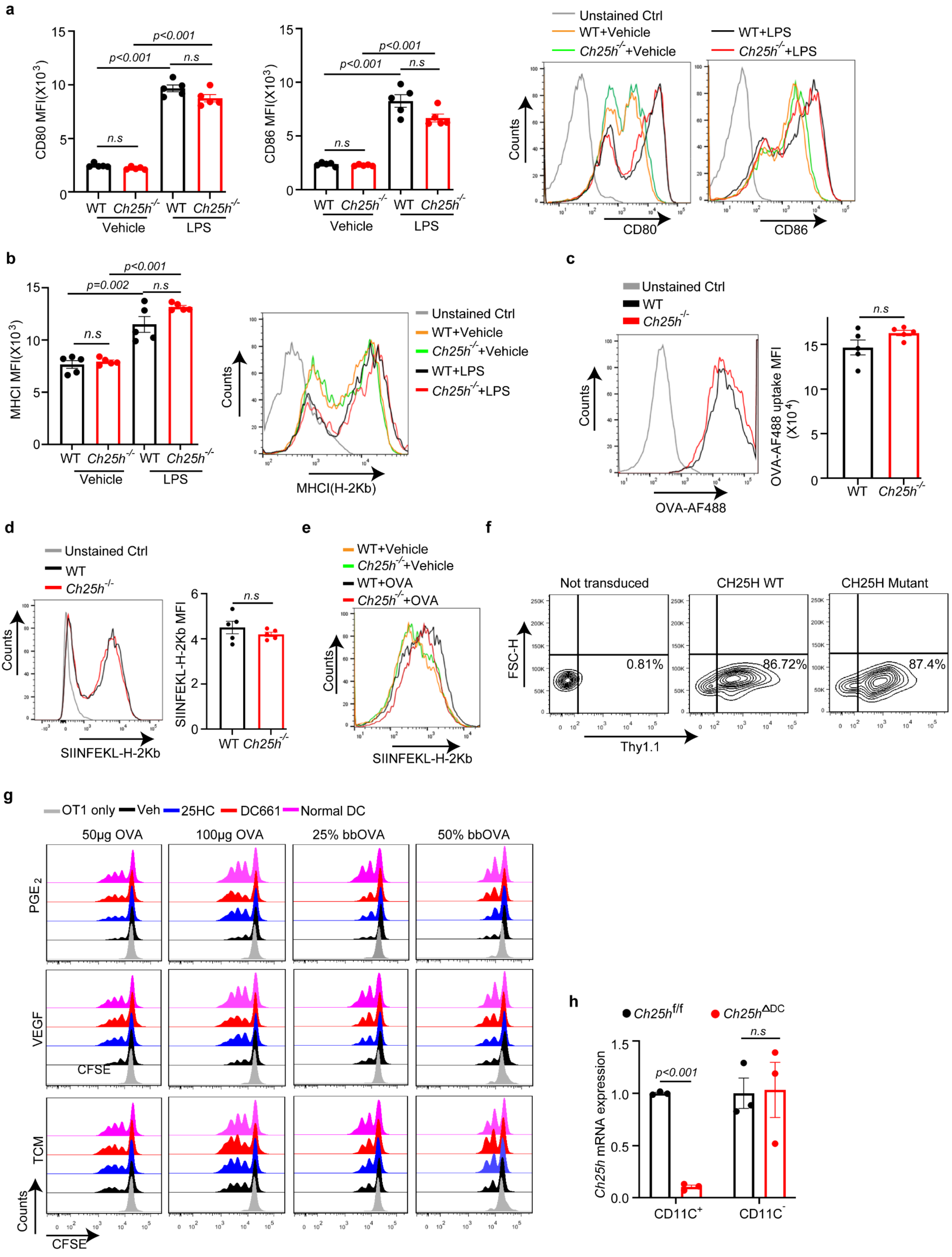

Supplementary Figure 3. CH25H expression in DCs enables efficient antigen cross-presentation otherwise inhibited by factors of tumor microenvironment

a. Flow cytometry analysis of CD80 and CD86 expression on DCs in WT and *Ch25h*$^{-/-}$ mice pretreated with or without LPS (100 ng/mL, 18 hr); *n* = 5 biologically independent samples. LPS, lipopolysaccharide; MFI, median fluorescence intensity.

b. Representative histograms and quantitative estimates of MHC I levels on DCs pre-treated with or without LPS (100ng/mL, 18 hr) from indicated mice. *n* = 5 biologically independent samples.

c. Uptake of OVA-AF488 (100 µg/ml, 1 hr) in DCs from WT and *Ch25h*$^{-/-}$ mice. *n* = 5 biologically independent samples.

d. Flow cytometry analysis of SIINFEKL-H-2Kb formation on DCs from indicated mice after treatment of OVA-derived short peptide (SIINFEKL, 1 µg/mL, 1 hr). n = 5 biologically independent samples

e. Representative histograms of experiments described in Figure 3a showing SIINFEKL-bound H-2kb expression on DCs from indicated mice after treatment of OVA protein (200 µg/mL, 18hr).

f. Relative expression of Thy1.1 in *Ch25h*$^{-/-}$ DCs transduced with MSCV-Ch25h-IRES-Thy1.1 retroviruses for expression of CH25H^{WT} or inactive CH25H^{H242,243Q} mutant.

g. Representative histograms of experiments described in Figure 3f-g. Proliferation was assessed by flow cytometry in CFSE-labeled OT-I T cells co-culture (10:1) for 72 hr with CD11c^{+} myeloid cells pulsed with complete OVA (50 µg and 100 µg, 6 hr) or beads-bound OVA (25% and 50%, 1 hr) pretreated with PGE_2 (10 ng/mL), VEGF (20 ng/mL) or TCM from LLC cells (75%, v/v)) for 24 hr with or without the treatment of 25HC (50nM) or DC661 (5µM) for 4 hr.

h. qPCR analysis of *Ch25h* mRNA levels in CD11c^{+} and CD11c^{+} splenic myeloid cells from indicated *Ch25h*$^{f/f}$ and *Ch25h* $^{\Delta DC}$ mice. *n* = 3 mice per group.

Data presented as mean±SEM. Statistical analysis was performed using 1-way ANOVA with Tukey’s multiple-comparison test (a and b) or 2-tailed Students’ t test (c, d and h). n.s., no significant. Source data are provided as a Source Data file.

Supplementary Fig.4

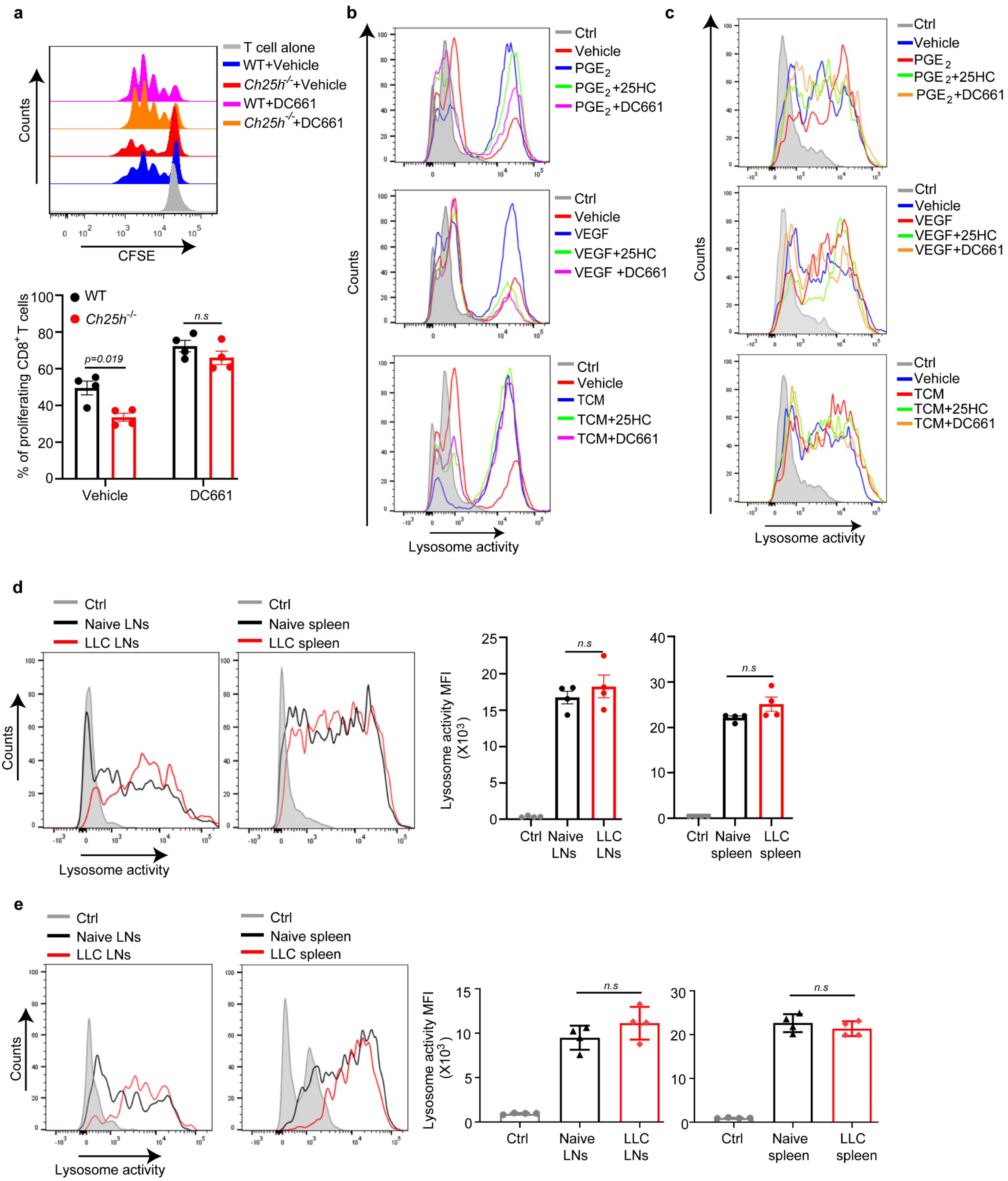

Supplementary Figure 4. CH25H expression in DCs acts to limit the lysosomal degradation otherwise induced by factors of tumor microenvironment

a. T cells proliferation was assessed by flow cytometry assessment of dilution of CFSE. CFSE-labeled OT-I cells were co-cultured (10:1 for 72hr) with indicated DCs pulsed with soluble OVA (200 μg/ml, 6 hr) pre-treated with or without DC661(5μM, 4 hr). n=4 biologically independent samples.

b. Representative histogram of 4a. Lysosome activity in human $CD14^+$ monocytes pretreated with PGE_2 (10 ng/mL), VEGF (20 ng/mL) or medium conditioned from A549 cells (75%, v/v)) for 24 hr with or without the treatment of 25HC (4μM) or DC661 (100nM).

c. Representative histogram of 4b. Lysosome activity in bone marrow $CD11c^+$ cells pretreated with PGE_2 (10 ng/mL), VEGF (20 ng/mL) or medium conditioned from LLC cells (75%, v/v)) for 24 hr with or without the treatment of 25HC (4μM) or DC661 (100nM).

d. Representative histogram of lysosome activity (DQ-OVA fluorescence) in total DCs from lung-draining LNs or spleen from naïve mice or LLC inoculated mice (*i.v.,* 1×10^6 cells/mouse, 2 weeks). n=4 biologically independent samples.

e. Representative of lysosome activity (DQ-OVA fluorescence) in $CD103^+CD11b^-$DCs of lung-draining LNs or spleen from naïve mice or LLC *i.v.* inoculated mice. *n* =4 biologically independent samples.

Data presented as mean±SEM. Statistical analysis was performed using 2-tailed Students' t test. n.s., no significant. Source data are provided as a Source Data file.

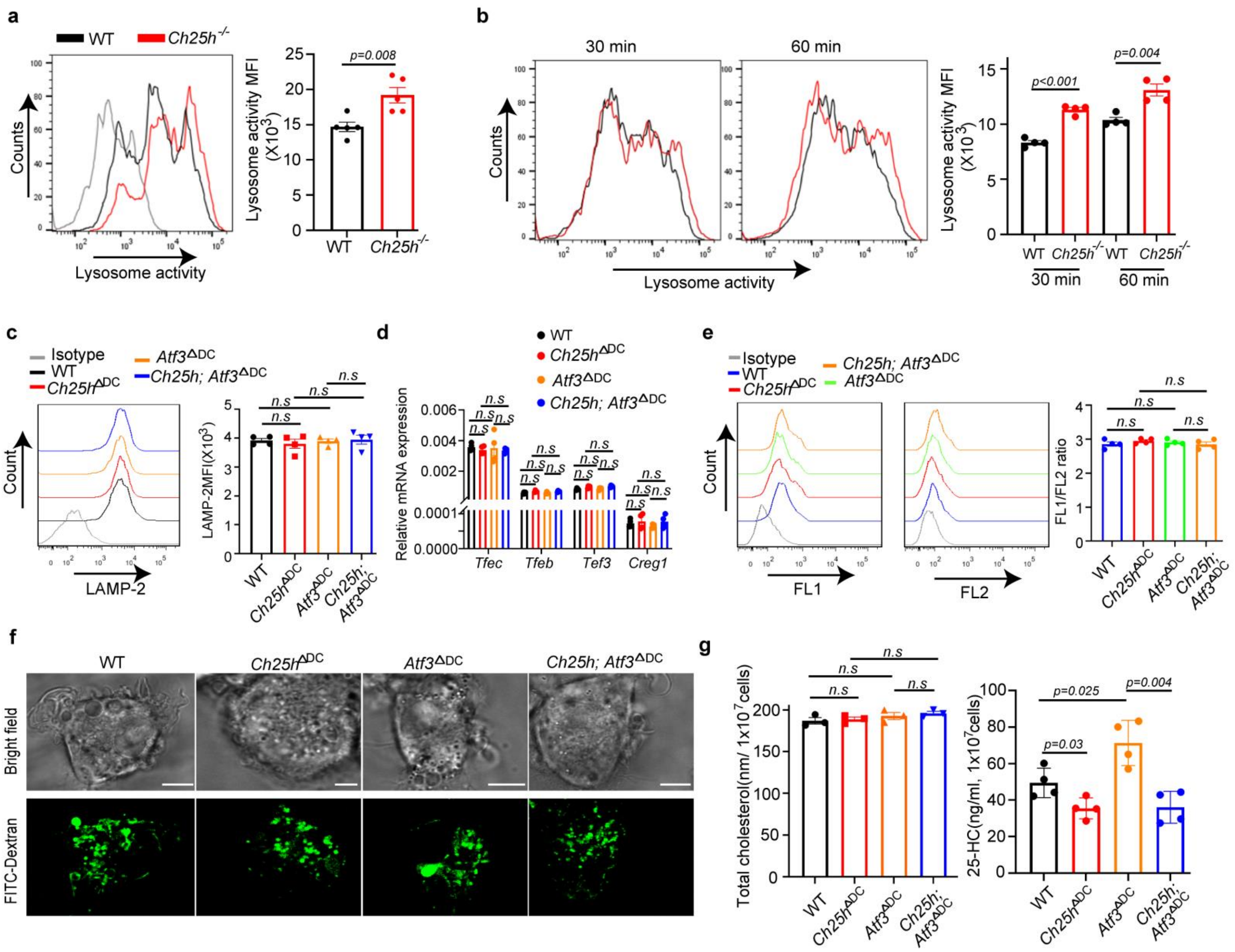
a
WT
Ch25h-/-
Counts
Lysosome activity
Lysosome activity MFI (X10^3)
p=0.008
WT
Ch25h-/-
b
30 min
60 min
Counts
Lysosome activity
Lysosome activity MFI (X10^3)
p<0.001
p=0.004
WT
Ch25h-/-
30 min
60 min
c
Isotype
WT
Ch25hΔDC
Atf3ΔDC
Ch25h; Atf3ΔDC
Count
LAMP-2
LAMP-2MFI(X10^3)
n.s
d
Relative mRNA expression
Tfec
Tfeb
Tef3
Creg1
e
FL1
FL2
FL1/FL2 ratio
f
Bright field
FITC-Dextran
g
Total cholesterol(nm/ 1x10^7cells)
25-HC(ng/ml, 1x10^7cells)
p=0.025
p=0.004
p=0.03

Supplementary Figure 5. CH25H regulates lysosomal proteolysis

a. Representative histograms and quantification of lysosome activity assessed by fluorescence of self-quenched substrate (BioVision, Cat. No. K448-50) in indicated DCs treated as indicated. n=5 biologically independent samples.

b. Representative histograms and quantification of lysosome activity assessed by fluorescence of DQ-OVA fluorescence in indicated DCs incubated with substrate for 30 min or 60 min. n=4 biologically independent samples.

c. Flow cytometry analysis of Lysosome marker (LAMP-2) in DCs isolated from spleen of indicated mice. n=4 biologically independent samples.

d. qPCR analysis of mRNA levels of indicated lysosome biogenesis-related genes in DCs isolated from spleens of indicated mice. n=4 biologically independent samples.

e. Flow cytometry analysis of lysosome pH in DCs isolated from spleen of indicated mice was performed as described in Methods. Quantification of the ratios of fluorescence intensity in FL1 versus FL2 channels is shown on the right. n=4 biologically independent samples.

f. Representative bright field and immunofluorescence images of distribution of FITC-Dextran (10kD, 0.1 mg/ml, Sigma, Cat. No. FD10S) in lysosomes of DCs isolated from spleens of indicated mice. Similar results were obtained from three independent experiments. Scale bar, 10μm.

g. ELISA-based analyses of total cholesterol (n=3 mice per group) and 25HC (n=4 mice per group) in DCs isolated from spleens of indicated mice.

Data presented as mean±SEM. Statistical analysis was performed using 2-tailed Students' t test. n.s., no significant. Source data are provided as a Source Data file.

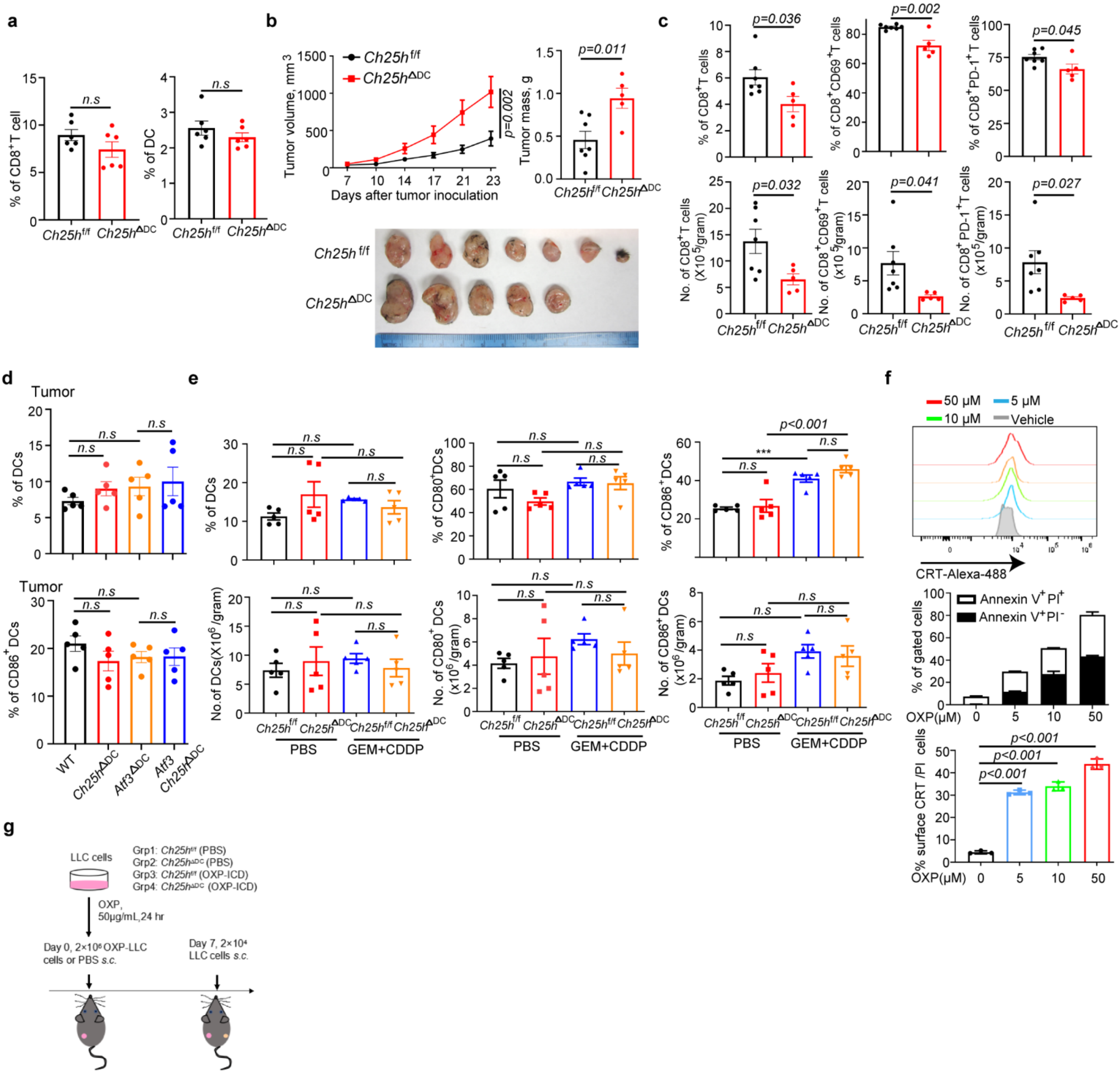
a
b
c
d
e
f
g
Ch25h f/f
Ch25h ΔDC
Tumor volume, mm3
Days after tumor inoculation
p=0.002
Tumor mass, g
p=0.011
p=0.036
p=0.002
p=0.045
p=0.032
p=0.041
p=0.027
n.s
Tumor
WT
Ch25hΔDC
Atf3ΔDC
Atf3 Ch25hΔDC
PBS
GEM+CDDP
p<0.001
50 μM
5 μM
10 μM
Vehicle
CRT-Alexa-488
Annexin V+PI+
Annexin V+PI-
% of gated cells
OXP(μM)
% surface CRT+/PI- cells
LLC cells
Grp1: Ch25hf/f (PBS)
Grp2: Ch25hΔDC (PBS)
Grp3: Ch25hf/f (OXP-ICD)
Grp4: Ch25hΔDC (OXP-ICD)
OXP, 50μg/mL,24 hr
Day 0, 2×10⁶ OXP-LLC cells or PBS s.c.
Day 7, 2×10⁴ LLC cells s.c.

Supplementary Figure 6. Downregulation of CH25H in DCs undermines the anti-tumor immunity and accelerates tumor growth.

a. Percentage of $CD8^+$ T cells and DCs in $CD45^+$ cells from naive WT and *Ch25h*$^{\Delta DC}$ mice, as determined by flow cytometry. *n* = 6 mice per group.

b. Growth of MC38 tumors after subcutaneous injection of 2.5×10^5 MC38 tumor cells into WT (n=7 mice) and *Ch25h*$^{\Delta DC}$ mice (n=5 mice). Representative images and quantification of MC38 tumor size and weight were taken and measured at day 24 after injection of MC38 cells.

c. Flow-cytometry determination of the percentage and quantitative estimates of intratumoral $CD8^+$ T cells and $CD69^+$, $PD1^+$ in $CD8^+$ T cells after *s.c.* inoculated with MC38 cells. *n* = 7 mice for *Ch25h*$^{f/f}$ group and n=5 for *Ch25h*$^{\Delta DC}$ groups.

d. Flow-cytometry determination of the percentage and CD86 expression of DCs isolated from LLC tumors in the experiment described in Figure 6e. n=5 mice for each group.

e. Flow-cytometry determination of the percentage and quantitative estimates of intratumoral DCs, $CD80^+$, $CD86^+$ DCs in *Ch25h*$^{f/f}$ and *Ch25h*$^{\Delta DC}$ mice with PBS or GEM^+CDDP treatment. *n* = 5 tumors in each treatment group.

f. Flow cytometry analysis of apoptotic LLC cells and percentage of externalized CRT on LLC cells after treatment of OXP (5-50μM, 24 hr). *n* = 3 per group. OXP, oxaliplatin; CRT, calreticulin.

g. Tumor vaccine treatment scheme in a therapeutic setting. Mice of indicated genotypes were immunized with the oxaliplatin-pretreated (50μM, 24 hr) LLC tumor cells (dose of 2×10^6 per mouse, *n* = 5 per genotype) on day 0. Another group for each genotype mice (*n* = 5 per genotype) received no vaccination at same day as no treatment control. On day 7 day, all mice were challenged with 1.5×10^4 non-treated LLC cells.

Data presented as mean±SEM. Statistical analysis was performed using 2-tailed Students' t test (a, b, c and f) or 1-way ANOVA with Tukey's multiple-comparison test (d and e) or 2-way ANOVA with multiple comparison (b). n.s., no significant. Source data are provided as a Source Data file.

Supplementary Fig.7

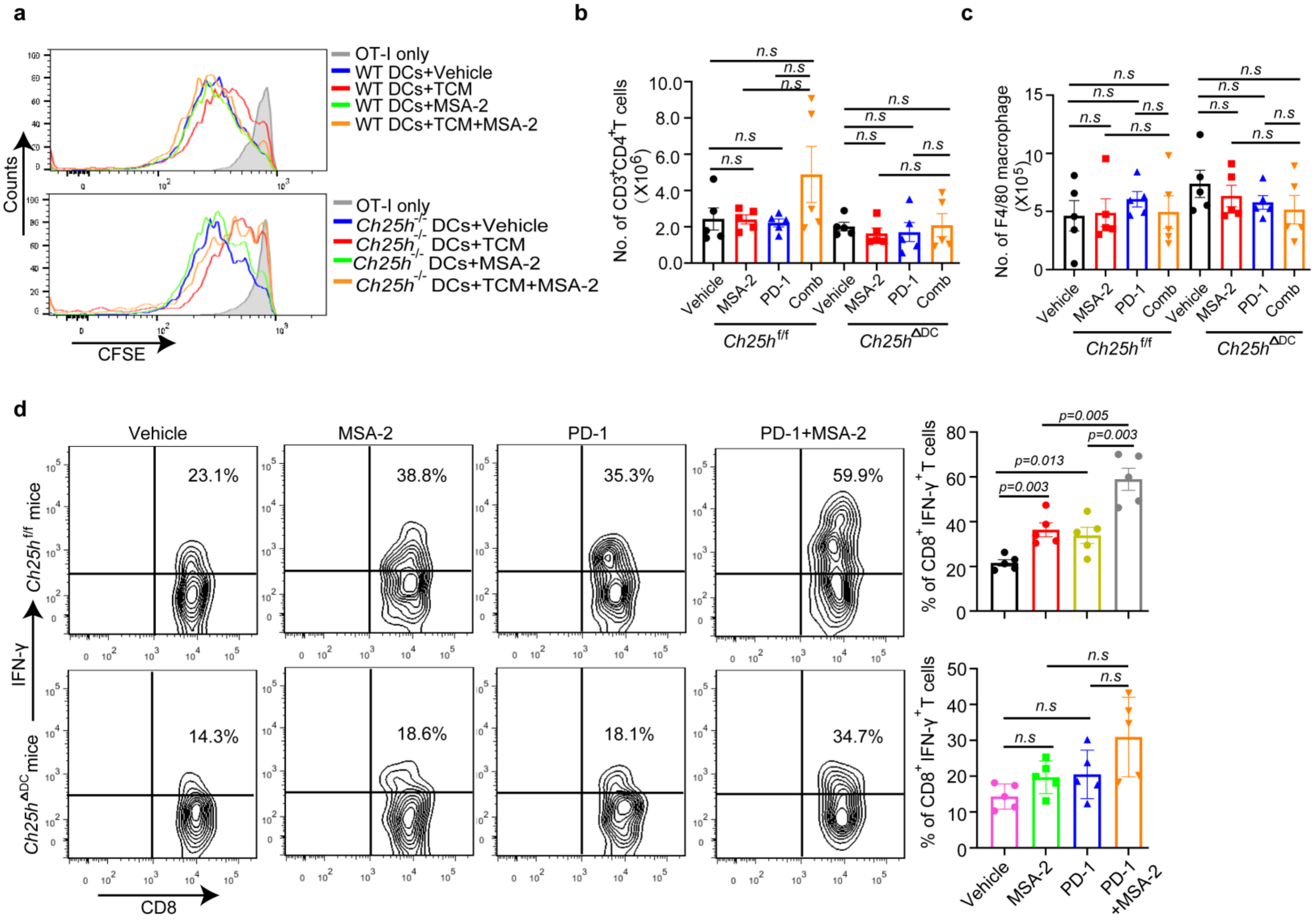

Supplementary Figure 7. STING agonist acts to maintain CH25H expression in DCs to increase intratumoral immune infiltration and elicit anti-tumor therapeutic effects.

a. Representative histogram for data shown in Figure 7c. Proliferation was assessed by flow cytometry in CFSE labeled OT-I T cells co-cultured (10:1) with OVA-pulsed (200 µg/mL, 6 hr) WT or *Ch25h*$^{-/-}$ BMDCs for 72 hr. BMDCs were pre-treated with medium conditioned from LLC cells (70%, v/v) for 20 hr with or without the treatment of MSA-2 (10µM) for 16 hr before OVA pulse.

b. Flow determination of number of $CD3^{+}CD4^{+}$ T cells in LLC tumors grown in *Ch25h*$^{f/f}$ or *Ch25h*$^{\Delta DC}$ mice with indicated treatment described in Figure 7d. n=5 mice per group.

c. Flow determination of number of F4/80^{+} macrophage in LLC tumors grown in *Ch25h*$^{f/f}$ or *Ch25h*$^{\Delta DC}$ mice with indicated treatment described in Figure 7d. n=5 mice per group

d. Flow determination of number of CD8^{+}IFN-γ^{+} T cells in LLC tumors grown in *Ch25h*$^{f/f}$ or *Ch25h*$^{\Delta DC}$ mice with indicated treatment described in Figure 7d. n=5 mice per group

Data presented as mean±SEM. Statistical analysis was performed using 2-tailed Students' t test. n.s., no significant. Source data are provided as a Source Data file.

Supplementary Fig.8

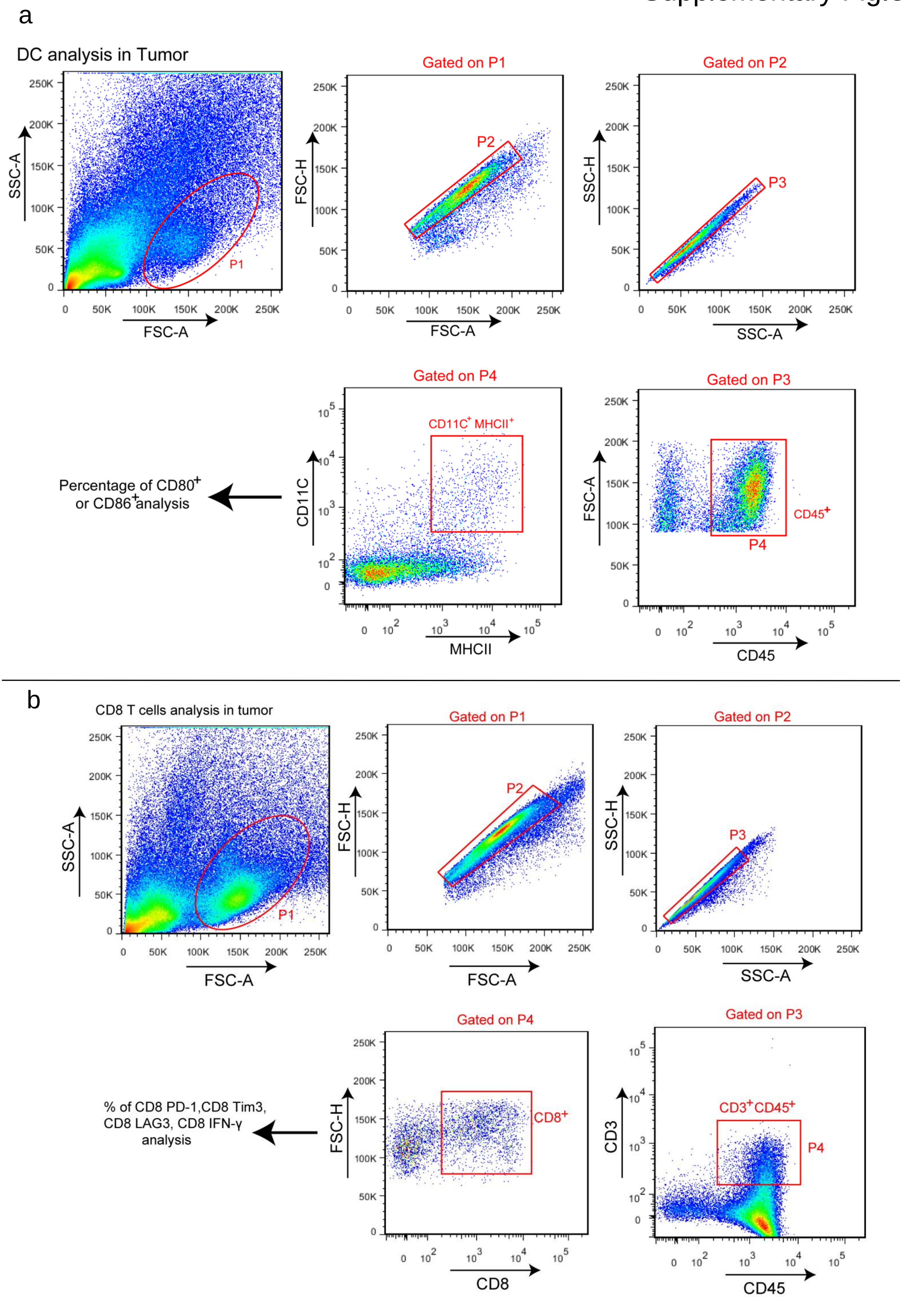

Supplementary Fig.8-continued

c

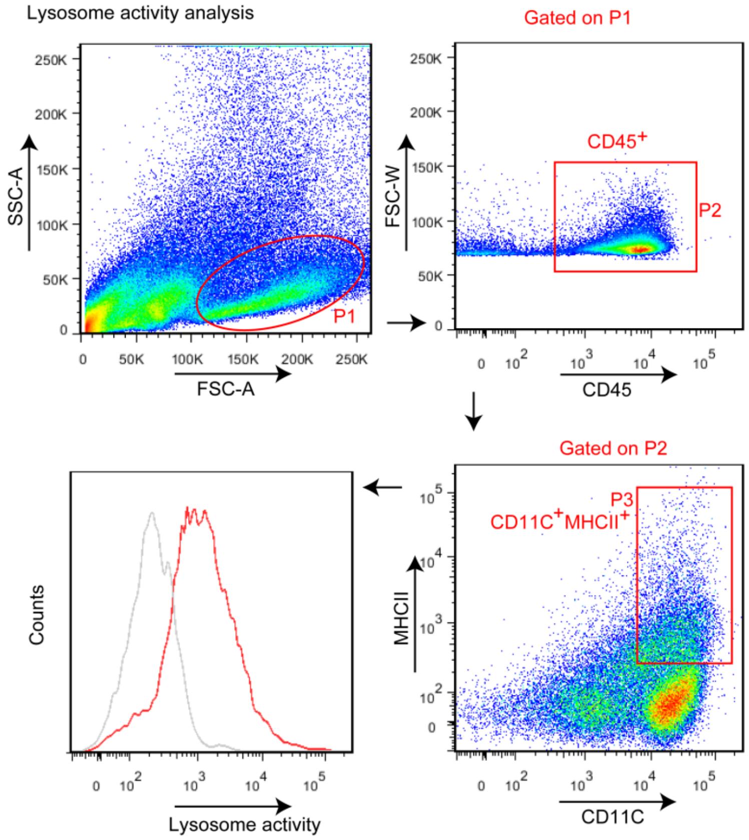

d

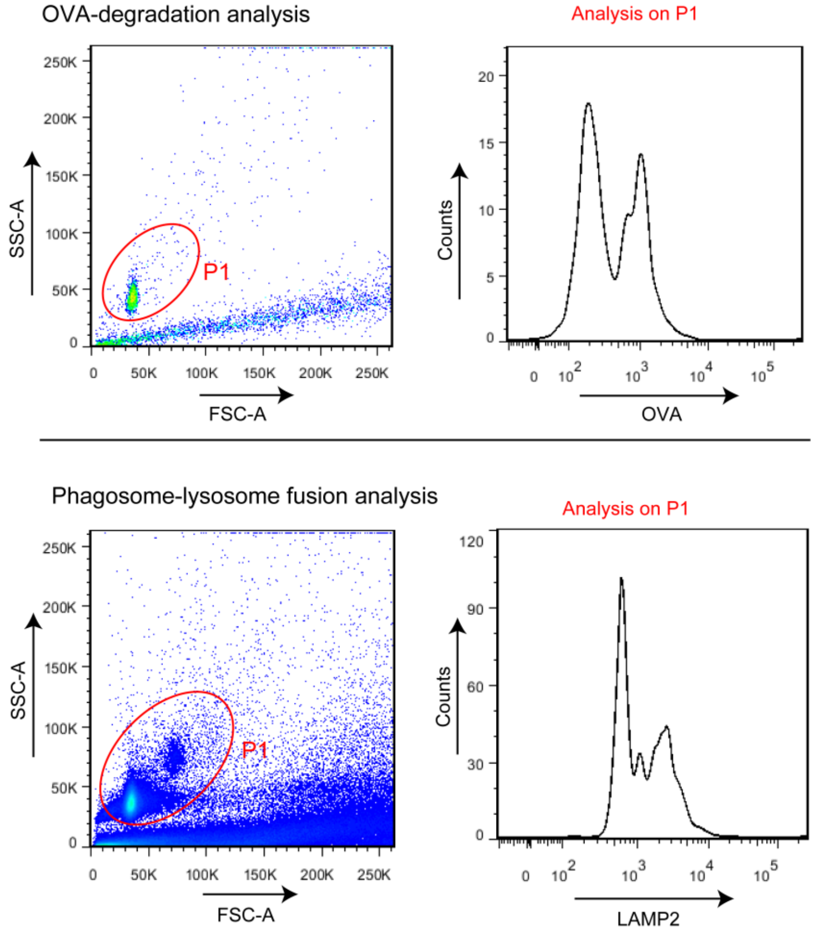

e

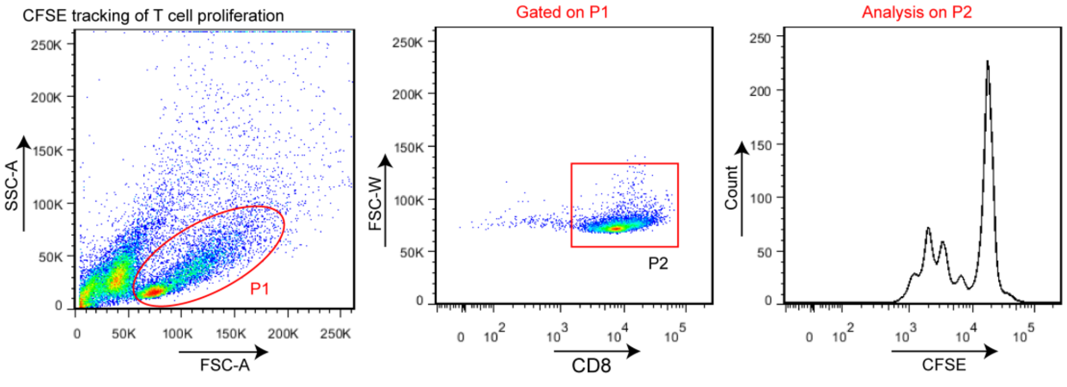

Supplementary Figure 8. Gating strategies of flow cytometry-related analysis.

a. Flow cytometry analysis of Dendritic cells in tumor microenvironment, refers to Fig S1d-e, Fig S6a and 6d-e.
b. Flow determination of intratumoral T cells, refers to Fig 1h, 6c, 6f, 6h, Fig 7g-h, Fig S1d, Fig S6a, 6d and 6e.
c. Flow determination of lysosome activity in vitro, refers to Fig 4, Fig 5, Fig S4b-e and Fig S5a-b.
d. Flow analysis of OVA degradation and phagosome-lysosome fusion in Fig 5f-g.
e. Flow analysis of T cell proliferation in Fig 3c, d, e and h.

Supplementary Table 1
List of oligonucleotides used for qPCR or mouse genotyping

**Oligonucleotides for qPCR:**	Source
Human *ATF3* Forward:<br>CCTGAACAGCGAAGTGTTGG	Sigma-Aldrich
Human *ATF3* Reverse:<br>TGGAGAACCCATGAGGTTTCAA	
Human *CH25H* Forward:<br>GCTACTCTTCGACATGGAGTTC	
Human *CH25H* Reverse:<br>CAGTTCCCAGACGCTCATATAC	
Human *GAPDH* Forward:<br>AGGGCTGCTTTTAACTCTGGT	
Human *GAPDH* Reverse:<br>CCCCACTTGATTTTGGAGGGA	
Mouse *ATF3* Forward:<br>TTACCGTCAACAACAGACCC	
Mouse *ATF3* Reverse:<br>TCAGCTCAGCATTCACACTC	
Mouse *CH25H* Forward:<br>TGCTACAACGGTTCGGAGC	
Mouse *CH25H* Reverse:<br>AGAAGCCCACGTAAGTGATGAT	
Mouse *β-actin* Forward:<br>AGAGGGAAATCGTGCGTGAC	
Mouse *β-actin* Reverse:<br>CAATAGTGATGACCTGGCCGT	
Mouse *Tfec* Forward:<br>GGTCTCACGGATGCTCCTTG	
Mouse *Tfec* Reverse:<br>TCCAGCGCATATCAGGATCATTA	
Mouse Tfeb Forward:<br>CCACCCCAGCCATCAACAC	
Mouse Tfeb Reverse:<br>CAGACAGATACTCCCGAACCTT	
Mouse Creg1 Forward:<br>GTGGCACTACTGGTGTCGC	
Mouse Creg1 Reverse:<br>CGCGCACCTCCTTTATTGTG	
Mouse Tef3 Forward:<br>TGCGTCAGCAGCTTATGAGG	
Mouse Tef3 Reverse:<br>AGACACGCCAATCACAGAGAT	
**Oligonucleotides (PCR) for genotyping:**	
Atf3$^{f/f}$ Forward:<br>TTCACTGCTAATAGCTCCTG	
Atf3$^{f/f}$ Reverse 1:	

TTCATAGCTCAGGGAACATCGG	
Atf3$^{f/f}$ Reverse 2: CAACTCCCTCTCCTCAAGTC	
Ch25h$^{f/f}$ Forward: AAGCCAAGTTAGTGCATTAGGGAA	
Ch25h$^{f/f}$ Reverse: CTATATGCTTGGCATGTGTGTCTTC	Sigma-Aldrich
CD11c-cre Forward: ACCTAGAGATGTTCGCGATTATCT	
CD11c-cre Reverse: ACCGTCAGTACGTGAGATATCTT	

Supplementary Table 2

List of antibodies for flow cytometry

**Target**	**Isotype**	**Conjugate**	**Clone**	**Company**
CD45	Rat IgG2b, κ	APC/cy7	30-F11	
CD45	Rat IgG2b, κ	APC	30-F11	
MHC II (IA/IE)	Rat IgG2b, κ	APC	M5/114.15.2	
MHC II (IA/IE)	Rat IgG2b, κ	PE/Cy7	M5/114.15.2	
CD11c	Armenian Hamster IgG	FITC	N418	
CD11c	Armenian Hamster IgG	PE	N418	
CD103	Armenian Hamster IgG	BV605	2E7	
CD80	Armenian Hamster IgG	FITC	16-10A1	
CD80	Armenian Hamster IgG	PE	16-10A1	
CD86	Rat IgG2a, κ	PE/CY7	GL-1	
CD86	Rat IgG2a, κ	APC	GL-1	
CD3	Rat IgG2b, κ	BV605	17A2	
CD3	Rat IgG2b,k	APC	17A2	
CD3	Rat IgG2b, κ	BV421	17A2	BioLegend
IFN-γ	Rat IgG1, κ	PE/Cy7	XMG1.2	
Granzyme B	Mouse IgG1, κ	Alexa Fluor 700	QA16A02	
CD279 (PD1)	Rat IgG2a, κ	BV605	29F.1A12	
CD69	Armenian Hamster IgG	FITC	H1.2F3	
Ki67	Rat IgG2a, κ	PE	16A8	
CD8	Rat IgG2a, κ	APC	53-6.7	
CD8	Rat IgG2a, κ	PE/Cy7	53-6.7	
CD8	Rat IgG2a, κ	Alexa Fluor 700	53-6.7	
CD8	Rat IgG2a, κ	APC/cy7	53-6.7	
CD8	Rat IgG2b, κ	FITC	YTS156.7.7	
CD8	Rat IgG2a, κ	PE	53-6.7	
CD4	Rat IgG2b, κ	Alexa Fluor700	GK1.5	

CD366	Rat IgG1, k	BV421	B8.2C12	
CD223	Rat IgG1, k	PE	C9B7W	
F4/80	Rat IgG2a, κ	BV421	BM8	
CD11b	Rat IgG2b, κ	Percp/cyanine5.5	M1/70	
Calreticulin	Rabbit IgG	Alexa Fluor 488	D3E6	Cell Signaling Technology
