# Supplementary material for: Tumor factors stimulate lysosomal degradation of tumor antigens and undermine their cross-presentation in lung cancer
Source: Nat Commun. 2022 Nov 4;13:6623. doi: 10.1038/s41467-022-34428-w (PMC9636202; doi:10.1038/s41467-022-34428-w)
Supplement: Supplementary file 2 — Reporting Summary [file 41467_2022_34428_MOESM2_ESM.pdf]

nature portfolio

Corresponding author(s): Serge Y. Fuchs.

Last updated by author(s): Oct 5, 2022

Reporting Summary

Nature Portfolio wishes to improve the reproducibility of the work that we publish. This form provides structure for consistency and transparency in reporting. For further information on Nature Portfolio policies, see our Editorial Policies and the Editorial Policy Checklist.

Statistics

For all statistical analyses, confirm that the following items are present in the figure legend, table legend, main text, or Methods section.

n/a	Confirmed	
☐	☒	The exact sample size (*n*) for each experimental group/condition, given as a discrete number and unit of measurement
☐	☒	A statement on whether measurements were taken from distinct samples or whether the same sample was measured repeatedly
☐	☒	The statistical test(s) used AND whether they are one- or two-sided<br>*Only common tests should be described solely by name; describe more complex techniques in the Methods section.*
☒	☐	A description of all covariates tested
☐	☒	A description of any assumptions or corrections, such as tests of normality and adjustment for multiple comparisons
☐	☒	A full description of the statistical parameters including central tendency (e.g. means) or other basic estimates (e.g. regression coefficient) AND variation (e.g. standard deviation) or associated estimates of uncertainty (e.g. confidence intervals)
☐	☒	For null hypothesis testing, the test statistic (e.g. *F*, *t*, *r*) with confidence intervals, effect sizes, degrees of freedom and *P* value noted<br>*Give P values as exact values whenever suitable.*
☒	☐	For Bayesian analysis, information on the choice of priors and Markov chain Monte Carlo settings
☒	☐	For hierarchical and complex designs, identification of the appropriate level for tests and full reporting of outcomes
☒	☐	Estimates of effect sizes (e.g. Cohen's *d*, Pearson's *r*), indicating how they were calculated

Our web collection on statistics for biologists contains articles on many of the points above.

Software and code

Policy information about availability of computer code

Data collection	no code was applied in data collection for this study
Data analysis	The data were analyzed using Flowjo V10.7.1 (BD), GraphPad Prism 8 (GraphPad Software Inc.), ImageJ1.5.3 (https://imagej.nih.gov/ij/), Image Studio Lite V5.2 and Software R.

For manuscripts utilizing custom algorithms or software that are central to the research but not yet described in published literature, software must be made available to editors and reviewers. We strongly encourage code deposition in a community repository (e.g. GitHub). See the Nature Portfolio guidelines for submitting code & software for further information.

Data

Policy information about availability of data

All manuscripts must include a data availability statement. This statement should provide the following information, where applicable:

- Accession codes, unique identifiers, or web links for publicly available datasets
- A description of any restrictions on data availability
- For clinical datasets or third party data, please ensure that the statement adheres to our policy

Gene expression microarray data that support the findings of this study have been previously reported Wolford et al., 2013b(see method section) and deposited in the NCBI Gene Expression Omnibus (GEO) repository under accession number GSE164611 (https://www.ncbi.nlm.nih.gov/geo/query/acc.cgi?acc=GSE164611) and analyzed by GENT2 public database. All other data supporting the findings of this study are available within the paper and its supplementary information files.

Field-specific reporting

Please select the one below that is the best fit for your research. If you are not sure, read the appropriate sections before making your selection.

☒ Life sciences ☐ Behavioural & social sciences ☐ Ecological, evolutionary & environmental sciences

For a reference copy of the document with all sections, see nature.com/documents/nr-reporting-summary-flat.pdf

Life sciences study design

All studies must disclose on these points even when the disclosure is negative.

Sample size	Sample size were determined based on extensive preliminary experiments and previous similar studies, basically each group minimum n= 3, and in most cases n=5, these numbers were sufficient for conducting statistical analysis.
Data exclusions	No data were excluded from analysis
Replication	Most of assays were carried out several times. Where indicated, experiments were conducted once using corresponding biological replicates per each experimental condition. These experiments were reliably reproduced at least once.
Randomization	Animal experiments were randomized. Mice with matched age and gender were randomly assigned into different groups for tumor growth analysis or treatment evaluation. Human samples were grouped based on preliminary qPCR analysis to determine whether expression of CH25H was decreased in tumor versus normal lung (group 1) or did not (group 2). Randomization for other experiments was irrelevant and impractical.
Blinding	The gene expression in CD14+ monocytes from human lung tumor required no intervention from the experimenter to isolating human monocytes and the one to run qPCR. As such, this portion of the experiment can be considered blinded in that the experimenters had no interaction with the patient sample collection aside from qPCR analysis. For the animal experiments, tumor size, weight and organs analysis were blinded by de-identifying samples. Blinding for other experiments was impractical.

Reporting for specific materials, systems and methods

We require information from authors about some types of materials, experimental systems and methods used in many studies. Here, indicate whether each material, system or method listed is relevant to your study. If you are not sure if a list item applies to your research, read the appropriate section before selecting a response.

Materials & experimental systems

n/a	Involved in the study	
☐	☒	Antibodies
☐	☒	Eukaryotic cell lines
☒	☐	Palaeontology and archaeology
☐	☒	Animals and other organisms
☐	☒	Human research participants
☒	☐	Clinical data
☒	☐	Dual use research of concern

Methods

n/a	Involved in the study	
☒	☐	ChIP-seq
☐	☒	Flow cytometry
☒	☐	MRI-based neuroimaging

Antibodies

Antibodies used	The following antibodies were used for flow cytometry:<br>anti-CD45 BioLegend, Cat#103112, Clone: 30-F11, dilution:200x<br>anti-CD45 BioLegend, Cat#103115, Clone: 30-F11, dilution:200x<br>anti-MHC II (IA/IE) BioLegend, Cat#107630, Clone: M5/114.15.2, dilution:200x<br>anti-MHC II (IA/IE) BioLegend, Cat#107613, Clone: M5/114.15.2,dilution:200x<br>anti-CD11c BioLegend, Cat#117305, Clone: N418, dilution:200x<br>anti-CD11c BioLegend, Cat#117307, Clone: N418, dilution:200x<br>anti-CD103 BioLegend, Cat#121433, Clone: 2E7, dilution:200x<br>anti-CD80 BioLegend, Cat#104707, Clone: 16-10A1, dilution:200x<br>anti-CD86 BioLegend, Cat#105012, Clone: GL-1, dilution:200x<br>anti-CD3 BioLegend, Cat#100237, Clone: 17A2, dilution:200x<br>anti-CD3 BioLegend, Cat#100227, Clone: 17A2, dilution:200x<br>anti-CD3 BioLegend, Cat#100236, Clone: 17A2, dilution:200x<br>anti-IFN-γ BioLegend, Cat#505825, Clone: XMG1.2, dilution:200x<br>anti-Granzyme B BioLegend, Cat#372221, Clone: QA16A02, dilution:200x<br>anti-CD279 (PD1) BioLegend, Cat#135220, Clone: 29F.1A12, dilution:200x

anti-CD69 BioLegend, Cat#104506, Clone: H1.2F3, dilution:200x
anti-Ki67 BioLegend, Cat#652404, Clone: 16A8, dilution:200x
anti-CD8 BioLegend, Cat#100711, Clone: 53-6.7, dilution:200x
anti-CD8 BioLegend, Cat#100721, Clone: 53-6.7, dilution:200x
anti-CD8 BioLegend, Cat#100730, Clone: 53-6.7, dilution:200x
anti-CD8 BioLegend, Cat#100714, Clone: 53-6.7, dilution:200x
anti-CD8 BioLegend, Cat#100705, Clone: 53-6.7, dilution:200x
anti-CD8 BioLegend, Cat#100708, Clone: 53-6.7, dilution:200x
anti-CD4 BioLegend, Cat#100430, Clone: GK1.5, dilution:200x
anti-CD366 BioLegend, Cat#134019, Clone: B8.2C12, dilution:200x
anti-CD223 BioLegend, Cat#125208, Clone: C9B7W, dilution:200x
anti-F4/80 BioLegend, Cat#123131, Clone: BM8, dilution:200x
anti-CD11b Biolegend, Cat#101228, Clone: M1/70, dilution:200x
anti-mouse CD86, BD, Cat#105013, Clone: GL-1, dilution: 200x
anti-Calreticulin Rabbit IgG Alexa Fluor 488, Cell Signaling Technology, Cat#62304S, RPID: AB_2799626,dilution:50x
anti-chicken OVA, Sigma, Cat# C6534, dilution: 5000X
anti-LAMP2, BD Biosciences, Cat# 550292, Clone:M3/84, dilution: 50X
Goat anti-rabbit Alexa Fluro 488, Invitrogen, Cat# A-11008, dilution: 500X
Goat anti-rat Alexa Fluro 594, Invitrogen, Cat# A-11007, dilution: 500X
FITC conjugated-CD107b monoclonal antibody, Invitrogen, Cat# 11-1072-81, Clone:eBioABL-93; dilution: 2000x

The following antibodies were used for Western Blot analysis:
anti-β-actin (Sigma, clone :AC-15, Cat# A1978, 0.5µg/ml), anti-Na, K-ATPase 1 (Cell Signaling Technology, Cat# 3010S, 1000x), anti-ATF3 (Cell Signaling Technology, clone: D2Y5W, Cat#33593,1000x), anti-CH25H Ab (Invitrogen, Cat#PA5-72349, RPID:AB_2718203, 1000x), IRDye 680RD donkey anti-rabbit secondary antibody (LI-COR Bioscience, Cat#926-68073,5000x)

Validation

All the antibodies are validated for the use of immunofluorescence and western blot analyses. Data are available on the manufacture`s website. The antibodies have been validated by the manufacturer or in this paper. No additional validation was carried out.

Eukaryotic cell lines

Policy information about cell lines

Cell line source(s)

Murine colon adenocarcinoma MC38 cells and Lewis lung carcinoma (LLC) cells were purchased form ATCC and maintained in DMEM (Gibco) supplemented with 10% FBS (HyClone) and 100 U/ml Penicillin-Streptomycin (Gibco). see method section. Phoenix packaging cells (from ATCC) were maintained at 37°C with 5% CO2 in RPMI-1640 including 10% heat-inactivated Fetal Bovine Serum (FBS), 50 U/ml penicillin-streptomycin and L-glutamine.

Authentication

Cells were authenticated by the vendor, no other authentications were conducted.

Mycoplasma contamination

All cell lines were tested negative for mycoplasma.

Commonly misidentified lines (See ICLAC register)

No commonly misidentified cell lines were used in the study.

Animals and other organisms

Policy information about studies involving animals; ARRIVE guidelines recommended for reporting animal research

Laboratory animals

All mice used in experiments were 6-8 weeks old males. Mice were housed in a specific pathogen free (SPF) condition (12 h light/12 h dark cycle, temperature 20-25°C) and had free access to water and chow (ANIMAL SPECIALTIES AND PROVISIONS, Lab diet 5010). Animal health status was routinely checked by qualified veterinarians. These mice were fed with regular chow and water ad libitum. Mice were maintained under specific-pathogen-free conditions in accordance with American Association for Laboratory Animal Science guidelines. WT (C57BL/6J, Stock No. 000664), Ch25h-/- (B6.129S6-Ch25htm1Rus/J, Stock No. 016263), OT-I (C57BL/6-Tg(TcraTcrb)1100Mjb/J, Stock No. 003831) and CD11c-cre (B6.Cg-Tg(Itgax-cre)1-1Reiz/J, Stock No. 008068) mice were purchased from The Jackson Laboratory. The conditional Ch25h allele (Ch25hf/f) was created as previously described (Lu et al., 2021b) and is in the process of being donated to the Jackson Laboratory (future Stock No. Stock No. 037647 C57BL/6-Ch25htm1.1Syfu/J). Atf3 flox allele (Atf3f/f) were described previously (Wolford et al., 2013b). All mice are in C57BL/6J background. CD11c-cre mice were crossed with Ch25hf/f mice or Atf3f/f mice to generate CD11c-cre::Ch25hf/f mice (Ch25hΔDC), CD11c-cre::Atf3f/f mice (Atf3ΔDC), or CD11c::Atf3f/f Ch25hf/f mice litter-mates.

Wild animals

The study did not involve wild animals.

Field-collected samples

The study did not involve field-collected samples.

Ethics oversight

All animal experiments were approved by the Institutional Animal Care and Use Committee of the University of Pennsylvania and were carried out in accordance with the IACUC guidelines.

Note that full information on the approval of the study protocol must also be provided in the manuscript.

Human research participants

Policy information about studies involving human research participants

Population characteristics	Detailed population characteristics of patients have been previously described in (Singhal et al., 2016; Singhal et al., 2019). A total of 18 patients with Stage I–III lung cancer, who were scheduled for surgical resection, were consented for tissue collection. All patients with LLC (including 14 males and 6 females) involved in this study were 51-84 old with informed consent and were provided with appropriate compensation. See manuscript.
Recruitment	All details on the recruitment have been previously described in (Singhal et al., 2016; Singhal et al., 2019). A portion of their tumor and non-cancerous adjacent lung tissue was used for research purposes at the Hospital of the University of Pennsylvania and The Philadelphia Veterans Affairs Medical Center after obtaining consents that had been approved by their respective Institutional Review Boards. These samples were precious - there was no exclusion of patients and hence no bias of any kind that could impact the results.
Ethics oversight	Tumor and non-cancerous adjacent lung tissue was used for research purposes at the Hospital of the University of Pennsylvania and The Philadelphia Veterans Affairs Medical Center after obtaining consents that had been approved by their respective Institutional Review Boards.

Note that full information on the approval of the study protocol must also be provided in the manuscript.

Flow Cytometry

Plots

Confirm that:

☒ The axis labels state the marker and fluorochrome used (e.g. CD4-FITC).

☒ The axis scales are clearly visible. Include numbers along axes only for bottom left plot of group (a 'group' is an analysis of identical markers).

☒ All plots are contour plots with outliers or pseudocolor plots.

☒ A numerical value for number of cells or percentage (with statistics) is provided.

Methodology

Sample preparation	Single cell suspensions from the indicated tissues were prepared as described method section in manuscript. Briefly, tumors or spleens were collected and incubated in dissociation solution with 2mg/ml Collagenase II (MP Biomedicals), or 1 mg/ml Collagenase IV (Roche) plus 100 μg/ml DNase I (Roche) for 1 h with continuous agitation. Cells were filtered by 70um cell strainer and resuspended with FACS buffer (PBS with 1% BSA, 1mM EDTA).<br>resuspended in staining buffer (PBS, 1% FBS, 1mM EDTA), followed by blocking of the Fc receptor and staining with the indicated antibodies for 30 minutes on ice in the dark.
Instrument	BD LSRFortessa and Canto
Software	FACS Diva software version v10(BD) were used to analyze flow data
Cell population abundance	Abundance of cell population was checked by flow cytometry.
Gating strategy	Gating for immune cells in tumor tissues: 1. Gate on FSC-A vs. SSC-A was set to include all cell populations except cell debris. 2. Gate on FSC-A vs. FSC-H to exclude doublets. 3.CD45+ cells were gated to rule out non-immune cell. 4. Gate on CD11c +MHCII+ to include DCs or CD3+CD8+ to include CD8+ T cells. 5. Further analyze protein levels in different cell subsets.

☒ Tick this box to confirm that a figure exemplifying the gating strategy is provided in the Supplementary Information.
